# Therapeutic activation of endothelial sphingosine‐1‐phosphate receptor 1 by chaperone‐bound S1P suppresses proliferative retinal neovascularization

**DOI:** 10.15252/emmm.202216645

**Published:** 2023-03-13

**Authors:** Colin Niaudet, Bongnam Jung, Andrew Kuo, Steven Swendeman, Edward Bull, Takahiro Seno, Reed Crocker, Zhongjie Fu, Lois E H Smith, Timothy Hla

**Affiliations:** ^1^ Department of Surgery, Vascular Biology Program, Boston Children's Hospital Harvard Medical School Boston MA USA; ^2^ Department of Ophthalmology, Boston Children's Hospital Harvard Medical School Boston MA USA

**Keywords:** neovascularization, Sphingosine‐1‐phosphate (S1P), vascular retinopathy, Vascular Biology & Angiogenesis

## Abstract

Sphingosine‐1‐phosphate (S1P), the circulating HDL‐bound lipid mediator that acts via S1P receptors (S1PR), is required for normal vascular development. The role of this signaling axis in vascular retinopathies is unclear. Here, we show in a mouse model of oxygen‐induced retinopathy (OIR) that endothelial overexpression of *S1pr1* suppresses while endothelial knockout of *S1pr1* worsens neovascular tuft formation. Furthermore, neovascular tufts are increased in *Apom* ^−/−^ mice which lack HDL‐bound S1P while they are suppressed in *Apom* ^*TG*^ mice which have more circulating HDL‐S1P. These results suggest that circulating HDL‐S1P activation of endothelial S1PR1 suppresses neovascular pathology in OIR. Additionally, systemic administration of ApoM‐Fc‐bound S1P or a small‐molecule Gi‐biased S1PR1 agonist suppressed neovascular tuft formation. Circulating HDL‐S1P activation of endothelial S1PR1 may be a key protective mechanism to guard against neovascular retinopathies that occur not only in premature infants but also in diabetic patients and aging people.

## Graphical abstract

The sphingosine‐1‐phosphate/sphingosine‐1‐phosphate receptor 1 axis can be activated to prevent aberrant retinal angiogenesis.

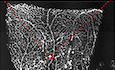

**The paper explained**

**Problem**

Pathological vascular leak and angiogenesis are key features shared by retinopathies such as wet age‐related macular degeneration, diabetic retinopathy, and retinopathy of prematurity. Vascular endothelial growth factor (VEGF), the main driver of abnormal retinal vasculature, is commonly targeted in such diseases. However, limitations of anti‐VEGF approaches have prompted the search for alternative therapeutic targets.

**Results**

We show that the endothelial sphingosine‐1‐phosphate receptor 1 (S1PR1) signaling protects from pathological retinal vascular leak and angiogenesis in mouse models. This was demonstrated by genetic overexpression of S1PR1 as well as pharmacological agonism of S1PR1 by systemic administration of a recombinant S1PR1 chaperone or a small‐molecule‐biased agonist. This signaling axis enhanced endothelial adherens junctions and mural cell coverage while suppressing vascular leak and abnormal growth of vascular tufts.

**Impact**

Our data suggest that the S1PR1 pathway is a novel therapeutic target in retinopathy of prematurity. We also suggest that the S1PR1 axis is easily amenable to activation through a systemic delivery of S1P chaperones or specific agonists. Finally, this study paves the way for further development of the therapeutic concept that adult vascular retinopathies can be targeted by agonism of endothelial S1PR1.

## Introduction

The retina is supplied by the specialized retinal and choroidal blood vessels. Pathological changes in these vascular beds occur either in adult diseases (i.e., diabetic retinopathy and age‐related macular degeneration) or in infants suffering from retinopathy of prematurity (ROP) and lead to decreased vision and blindness. A common theme in such diseases is pathological angiogenesis (neovascularization) that follows tissue ischemia (Kermorvant‐Duchemin *et al*, 2010). Inhibition of vascular endothelial growth factor (VEGF)‐driven pathological angiogenesis has been the mainstay of therapeutic approaches for retinal neovascular diseases. However, this approach has limitations due to the requirement for intravitreal injection of VEGF‐neutralizing agents, the involvement of VEGF signaling in the maintenance of the choroidal vasculature and retinal neurons, and low efficacy in a subset of patients. Such constraints have prompted the search for alternative therapeutic approaches, particularly a treatment that inhibits neovascularization without affecting normal vascular development (Saint‐Geniez *et al*, 2009; Kurihara *et al*, 2012; Bakri *et al*, 2014; Usui‐Ouchi & Friedlander, 2019).

Sphingosine‐1‐phosphate (S1P), a bioactive sphingolipid, is required for vascular development (Mizugishi *et al*, 2005). In endothelial cells in developing vessels, S1P acts through G‐protein‐coupled receptor S1PR1 to restrict VEGF‐induced angiogenesis and promote vascular stability in multiple vascular beds (Chae *et al*, 2004; Ben Shoham *et al*, 2012; Gaengel *et al*, 2012; Jung *et al*, 2012). We recently showed that S1PR1‐induced adherens junction formation suppresses VEGF‐induced JunB transcription factor in the nascent vascular network, which allows *Wnt*‐dependent organotypic specialization events to take place, a key mechanism in the normal maturation of the retinal vessels (Yanagida *et al*, 2020). Even though S1P is produced locally, circulating S1P is bound to the chaperone ApoM on HDL particles (Christoffersen *et al*, 2011).

How S1P signaling impacts proliferative retinopathies is not well understood. In oxygen‐induced retinopathy (OIR), a mouse model that mimics ROP and other proliferative retinopathies (Smith *et al*, 1994), sphingosine kinase‐2 gene (*Sphk2*), and *S1pr2* KO mice (Skoura *et al*, 2007; Eresch *et al*, 2018), or mice treated with a blocking antibody against S1P (Xie *et al*, 2009) were protected from pathological neovascularization. These results implicated that locally produced S1P activation of S1PR2 exacerbates the pathological changes in ROP. However, in humans, circulating S1P is decreased in severe ROP (Nilsson *et al*, 2021) and endothelial S1PR1 is known to protect from endothelial injury in many disease models (Cartier & Hla, 2019). To clarify the role of S1P in OIR, we studied mutant mice that lack or overexpress endothelial S1PR1 as well as circulating Apolipoprotein M (ApoM) in the mouse OIR model. Our results show that circulating HDL‐bound S1P and endothelial S1PR1 suppress pathological neovascular tuft formation by inhibiting abnormal vascular leakage while enabling pericyte ensheathment. Systemic administration of recombinant ApoM‐Fc‐bound S1P or a small‐molecule G_i_‐biased agonist of S1PR1 reduced neovascular tuft formation in OIR, suggesting that this pathway is therapeutically targetable in neovascular retinal pathologies.

## Results and Discussion

### Endothelial S1PR1 suppresses neovascular tuft formation in OIR

**Figure EV1:**
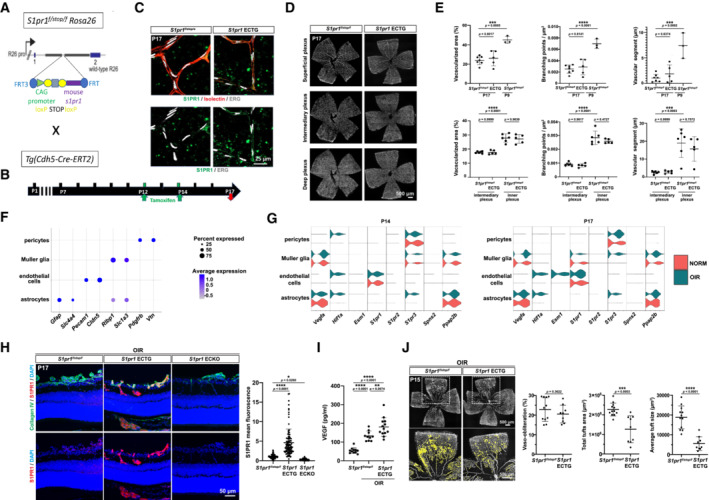
S1PR1 signaling in retinal EC inhibits neovascularization in OIR Schematic representation of the breeding strategy to establish *S1pr1* ECTG: females carrying the *(ROSA)26Sortm1(CAG‐S1pr1)* transgene were crossed to *Cdh5‐Cre* ^*ERT2*^ Cre males.Strategy to induce expression of S1PR1 in post‐natal endothelium. Pups were given tamoxifen at P12 and P14, and retinas were analyzed at P17.Flat‐mounted retinas from OIR *S1pr1* ^*f/stop/f*^ and *S1pr1* ECTG pups at P17. High‐magnification pictures of superficial capillaries showing S1PR1 induction in the *S1pr1* ECTG.Flat‐mounted retinas from OIR *S1pr1* ^*f/stop/f*^ and *S1pr1* ECTG pups at P17. Retinal vasculature parameters were stained with isolectin and images of superficial (top panel), intermediary (middle panel), and deep (lower panel) plexuses are shown.Quantification of morphometric parameters in between retinas from *S1pr1* ^*f/stop/f*^ and *S1pr1* ECTG. Retinas from P9 S1pr1^f*/stop/f*^ mice were used as controls.Dot plot representing expression level and frequency of cell types markers among non‐neuronal retinal cells at P14 and P17 in OIR.Volcano plot showing expression level and frequency of OIR‐induced genes (left) and S1P‐related genes (right) among non‐neuronal retinal cells at P14 (left) and P17 (right) in OIR. *S1pr4* and *S1pr5* expression was limited to a low number of endothelial cells.Cross sections from OIR *S1pr1* ^*f/stop/f*^, *S1pr1* ECTG, and *S1pr1* ECKO P17 pups stained for S1PR1 (red). Blood vessels are delineated by collagen IV (green) and nuclei are stained with Hoechst (blue) (left). Quantification of associated S1PR1 fluorescence inside blood vessels (right).VEGF level in P17 retinas quantified by ELISA. Retinas from normoxic *S1pr1* ^*f/stop/f*^ animal (*n* = 10) or OIR *S1pr1* ^*f/stop/f*^ (*n* = 10) and *S1pr1* ECTG pups (*n* = 13) at P17 were used.Flat‐mounted retinas from OIR *S1pr1* ^*f/stop/f*^ and *S1pr1* ECTG pups at P15. Blood vessels are stained with isolectin (left). Avascular area, total neovascular tuft area, and average neovascular tuft size are quantified (right). Data information: Data in (E, H and I) were analyzed by ANOVA test, and in (J) by one‐tailed Student's *t*‐test. Data are expressed as mean ± SD. A minimum of three pups per group were analyzed.

To study the role of S1PR1 in the OIR model, EC *S1pr1* transgene (*S1pr1* ECTG) was induced by tamoxifen administration from post‐natal days 12 to 14 (P12–14). This regime did not influence the normal development and maturation of retinal vascular plexuses (Fig EV1A–E). Analysis of droplet‐based single‐cell RNA‐sequencing (scRNA‐seq) data from wild‐type (WT) P17 retina (Binet *et al*, 2020) revealed that *S1pr1* is expressed at the highest level in EC, while lower expression was observed in the Müller glial cells. OIR challenge did not alter *S1pr1* expression at P14 or P17 (Fig EV1F and G). *S1pr3* was expressed by retinal pericytes, whereas *S1pr2* expression was barely detectable in the retinal cells. The presence of S1PR1 protein in capillaries and neovascular tufts was confirmed by immunofluorescence staining of retinal sections post‐OIR. While *S1pr1* ECTG showed the strongest endothelial S1PR1 expression (~4‐fold induction), *S1pr1* ECKO mice were essentially devoid of S1PR1 immunoreactivity in EC (Fig EV1H), which validated our S1PR1 protein detection method.

**Figure 1:**
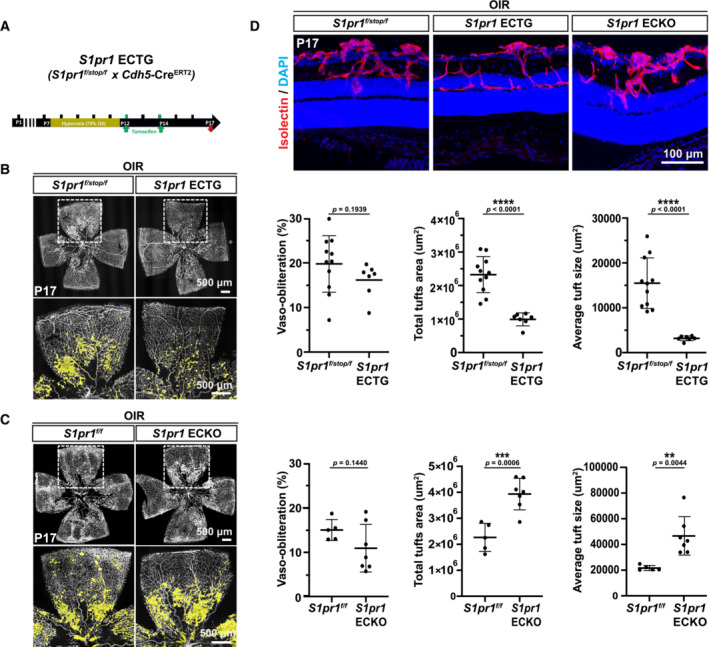
Endothelial S1PR1 signaling suppresses pathological neovascular tufts in OIR Strategy to induce S1PR1 expression in the endothelium post‐OIR. *S1pr1* ECTG pups were exposed to 75% oxygen from P7 to P12. Upon return to normoxia, S1PR1 expression was induced by tamoxifen injection at P12 and P14, and retinas were analyzed at P17.Flat‐mounted retinas from OIR *S1pr1* ^*f/stop/f*^ and *S1pr1* ECTG pups at P17. Blood vessels are stained with isolectin (left) and pathological neovascular tuft area is highlighted in yellow. Avascular area and total and average neovascular tuft areas are quantified (right)Flat‐mounted retinas from OIR *S1pr1* ^*f/f*^ and *S1pr1* ECKO pups, at P17. Blood vessels are stained with isolectin and neovascular tufts are highlighted in yellow. Avascular area and total and average neovascular tuft areas are quantified.Cross sections from OIR *S1pr1* ^*f/stop/f*^, *S1pr1* ECTG, and *S1pr1* ECKO P17 pups. Blood vessels are stained with isolectin (red) and nuclei with Hoechst (blue). Data information: Data in (B and C) were analyzed by one‐tailed Student's *t*‐test. Data are expressed as mean ± SD. A minimum of five pups were analyzed per group.

*S1pr1* ^*f/stop/f*^ and *S1pr1* ECTG mice were placed in hyperoxic chamber from P7 to P12, and returned to a normoxic environment (relative hypoxia because of loss of formed retinal vessels during hyperoxia) from P12 to P17. In this model, maximum neovascularization is seen at P17, and vascular tufts resolve from P17 to P21. *S1pr1* overexpression from P12 to P14 resulted in a strong decrease in the total area and individual size of neovascular tufts in P17 retinas (Fig 1A and B). This was also observed in cross‐sections (Fig 1D). Revascularization toward the central retina was similar in *S1pr1* ^*f/stop/f*^ and *S1pr1* ECTG mice. In fact, VEGF level in the retinal extracts during OIR was slightly higher in the *S1pr1* ECTG retinas (Fig EV1I). The reduction in neovascular tufts in *S1pr1* ECTG retinas was observed as early as P15 and sustained until P17 (Fig EV1J), suggesting that S1PR1 suppresses the formation of neovascular lesions even in the presence of VEGF.

In contrast, genetic inactivation of *S1pr1* in the endothelium (*S1pr1* ECKO) at P12‐14 resulted in an increase in the total area of neovascular tufts as well as an increased size of individual tufts at P17 in the OIR model (Fig 1C and D). Similar to *S1pr1* ECTG, the avascular area in the ECKO was not significantly altered. Overall, our results using gain‐ and loss‐of function genetic models suggest that S1PR1 signaling in EC restrains the development of pathological angiogenesis by reducing VEGF‐induced neovascular tufts in the OIR model.

### Endothelial intrinsic function of S1PR1 suppresses vascular leakage, enhances pericyte coverage, and resolves neovascular tufts

**Figure 2:**
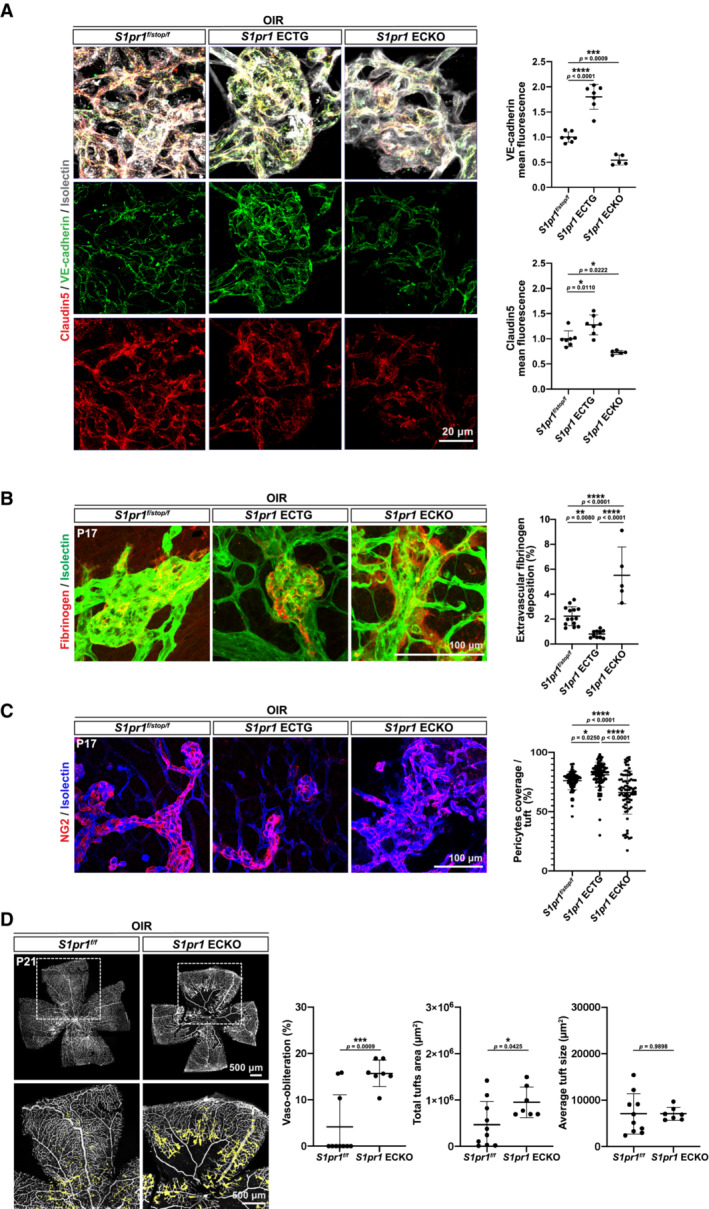
Endothelial S1PR1 enhances junctions while inhibiting vascular leakage and pericyte loss in OIR Retinal flat mounts from OIR *S1pr1* ^*f/stop/f*^ or *S1pr1* ECTG or ECKO P17 pups. High‐magnification view of neovascular tufts stained for VE‐cadherin (green) and Claudin‐5 (red). Junctional levels of VE‐cadherin and Claudin‐5 were quantified as described, for a minimum of five animals per genotype.Retinal flat mounts from OIR *S1pr1* ^*f/stop/f*^ or *S1pr1* ECTG or ECKO P17 pups. Sites of vascular leakage were assessed by staining for fibrinogen (red), and blood vessels are delineated by isolectin (green). Extravascular fibrinogen was quantified as described for a minimum of five animals per genotype.Pericytes stained by NG2 on the surface of isolectin‐positive neovascular tufts at P17 post‐OIR (left). EC‐associated pericytes in neovascular tufts were quantified (right). A total of 70 tufts from three different animals were assessed.Flat‐mounted retinas from OIR *S1pr1* ^*f/f*^ and S1pr1 ECKO pups at P21 stained with isolectin. Avascular area and total and average neovascular tuft areas are quantified. A minimum of seven pups were analyzed per group. Data information: Data in (A–C) were analyzed by ANOVA, and in (D) by one‐tailed Student's *t*‐test. Data are expressed as mean ± SD.

**Figure EV2:**
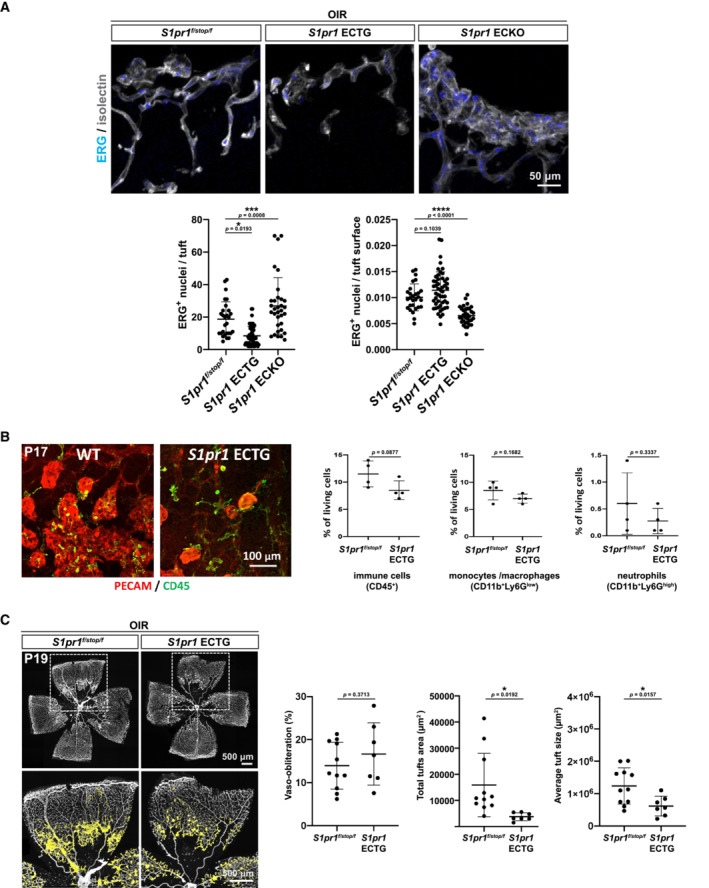
Neovascular tuft phenotypes in *S1pr1* ECTG at P17 Flat‐mount view of neovascular tufts from *S1pr1* ^*f/stop/f*^, *S1pr1* ECTG, or ECKO at P17, stained for endothelial nuclei (ERG), blood vessel (isolectin), and associated endothelial nuclei quantification.Flat‐mount view of neovascular tufts from *S1pr1* ^*f/stop/f*^ or *S1pr1* ECTG at P17, stained for immune cells (CD45), blood vessels (isolectin) (left), and FACS on retinal single‐cell preparations (right). Quantification of immune cells by FACS in OIR retinas from *S1pr1* ^*f/stop/f*^ and *S1pr1* ECTG at P17. Total immune cells (CD45 positive, left panel), monocytes/macrophages (CD11b positive, middle panel), and neutrophils (CD11b and Ly6G double positive, right panel) are presented.Flat‐mounted retinas from OIR *S1pr1* ^*f/stop/f*^ and *S1pr1* ECTG pups at P19 stained with isolectin. Avascular area and total and average neovascular tuft areas are quantified. Data information: Data in (A) were analyzed by ANOVA, and in (B, C) by one‐tailed Student's *t*‐test. Data are expressed as mean ± SD. A minimum of three pups per group were analyzed.

In OIR, dysregulated VEGF signaling induces vascular leakage and formation of vascular tufts, a major cause of vision loss. Staining of vascular tufts with adherens junction marker VE‐cadherin indicated increased junctional localization of VE‐cadherin in *S1pr1* ECTG, suggesting that EC S1PR1 induced adherens junctions (Fig 2A). However, tight junction marker Claudin‐5 increase was less marked. In addition, fibrinogen staining denotes decreased vascular leak in *S1pr1* ECTG compared to controls (Fig 2B). Indeed, fibrinogen was largely confined to the endothelial lumen in *S1pr1* ECTG tufts. In *S1pr1* ECKO tufts, VE‐cadherin staining was reduced while fibrinogen showed a diffuse extravascular pattern. We also assessed pericyte coverage on the tufts. In *S1pr1* ECTG, NG2‐positive pericytes covered most of the surface of the neovascular tufts. In contrast, the pericyte coverage was much sparser in *S1pr1* ECKO tufts, which were frequently lacking secondary processes and were detached from EC of neovascular tufts (Fig 2C). Vascular tufts in *S1pr1* ECTG animals contained fewer EC than the *S1pr1* ^*f/stop/f*^ counterparts, suggesting that S1PR1 limits EC proliferation despite high VEGF levels (Fig EV2A). These data indicate that S1PR1 signaling suppresses VEGF signaling in EC resulting in decreased permeability, junctional integrity, and increased pericyte coverage. Mechanisms involved may be similar to embryonic development in which S1PR1 signaling in the EC induces adherens junction assembly (Lee *et al*, 1999) and pericyte/EC interactions (Paik *et al*, 2004). Together, we show that in OIR, despite hypoxia and abnormal VEGF signaling, EC S1PR1 suppresses neovascular tuft formation and helps resolve pathological vascular lesions.

Recent reports proposed that neutrophils are involved in the resolution of neovascularization in the OIR model (Binet *et al*, 2020). We examined the number of retinal leukocytes, macrophages, and neutrophils, which were similar in *S1pr1* ECTG retinas versus *S1pr1* ^*f/stop/f*^ counterparts (Fig EV2B). The rate of neovascular tuft resolution was comparable regardless of S1PR1 expression level when comparing *S1pr1* ECTG, ECKO, and *S1pr* ^*f/f*^ at P19‐21. *S1pr1* ECKO tufts, which are greatest at P17, are still evident at P21, whereas *S1pr* ^*f/f*^ tufts had mostly resolved. Additionally, neovascularization was greater in *S1p1* ^*f/stop/f*^ than in *S1pr1* ECTG counterparts at P19 (Figs 2D and EV2C). Together, these results indicate that EC S1PR1 signaling regulates neovascular tuft formation and maintenance rather than resolution processes.

### Therapeutic activation of S1PR1 suppresses neovascular retinopathy

**Figure 3:**
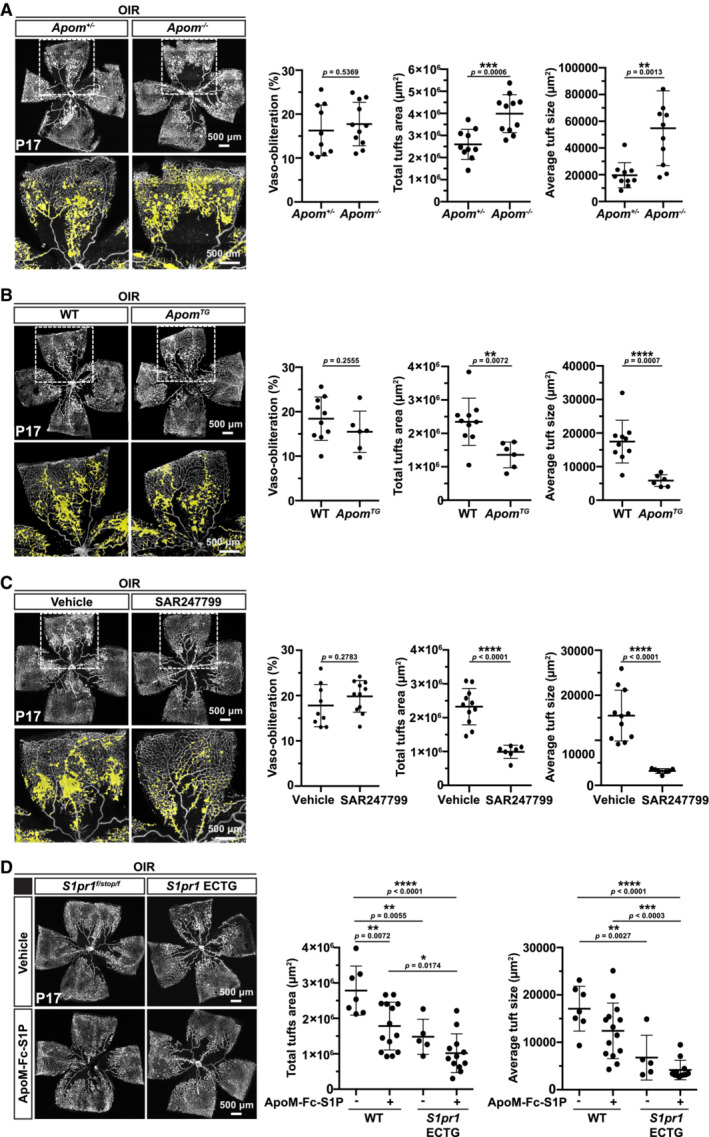
Systemic administration of S1PR1 agonists as a therapeutic strategy in retinopathy Flat‐mounted retinas from OIR *Apom* ^+/−^ and *Apom* ^−/−^ pups at P17, stained with isolectin. Avascular area and total and average neovascular tuft areas are quantified (*n* = 10 pups/group).Flat‐mounted retinas from OIR WT and *Apom* ^*TG*^ pups at P17, stained with isolectin. Avascular area and total and average neovascular tuft areas are quantified (*n* = 7 pups/group).Flat‐mounted retinas from SAR247799‐treated, OIR WT pups at P17, stained with isolectin. Avascular area and total and average neovascular tuft areas are quantified (*n* = 10 pups/group).Flat‐mounted retinas from ApoM‐Fc‐S1P‐treated, OIR *S1pr1* ^*f/stop/f*^, or *S1pr1* ECTG pups at P17, stained with isolectin. Avascular area and total and average neovascular tuft areas are quantified (*n* = 6 pups/group). Data information: Data in (A–C) were analyzed by one‐tailed Student's *t*‐test and in (D) by ANOVA. Data are expressed as mean ± SD.

To address whether S1P ligand bioavailability impacts neovascularization in OIR, we exposed mice in which the HDL‐bound S1P is absent—that is, *Apom* knockout (*Apom* ^−/−^) mice to OIR (Christoffersen *et al*, 2011). P17 retinas from *Apom* ^−/−^ exhibited enhanced pathological neovascularization, compared to those from littermate controls (Fig 3A). Conversely, mice constitutively overexpressing ApoM (*Apom* ^*TG*^), which contain 7–9 times more plasma ApoM than WT mice, had less retinal neovascular tuft formation (Fig 3B). These data suggest that circulating HDL‐bound S1P suppresses neovascular lesion formation in the OIR model. These data also imply that administration of chaperone‐bound S1P or pharmacological mimics may be an effective therapeutic strategy in retinopathy.

HDL‐S1P selectively activates EC S1PR1 and does not influence lymphocyte trafficking from secondary lymphoid organs and thymus (Wilkerson *et al*, 2012; Christensen *et al*, 2016; Swendeman *et al*, 2017). This is thought to be achieved via Gi‐biased signaling of S1P on EC S1PR1 with minimal ß‐arrestin activation, which downregulates the S1PR1 (Galvani *et al*, 2015; Poirier *et al*, 2020). Moreover, the large size of HDL‐S1P likely prevents it from modulating lymphocyte S1PR1 in secondary lymphoid organs or thymus, which is needed for suppression of lymphocyte egress and trafficking. Recently, a small‐molecule G_i_‐biased agonist of S1PR1 was shown to activate EC S1PR1 and suppress inflammatory processes without inducing lymphopenia. In humans, this compound, SAR247799, enhanced NO‐dependent cardiac reperfusion in diabetic subjects in a human phase 1 clinical study without inducing lymphopenia (Bergougnan *et al*, 2021). SAR247799 was injected for 5 consecutive days into pups post‐oxygen exposure. We found that systemic SAR247799 administration significantly reduced neovascular tuft formation (Fig 3C). This suggests that G_i_‐biased agonism of S1PR1 leads to the suppression of neovascular tuft formation in OIR.

**Figure EV3:**
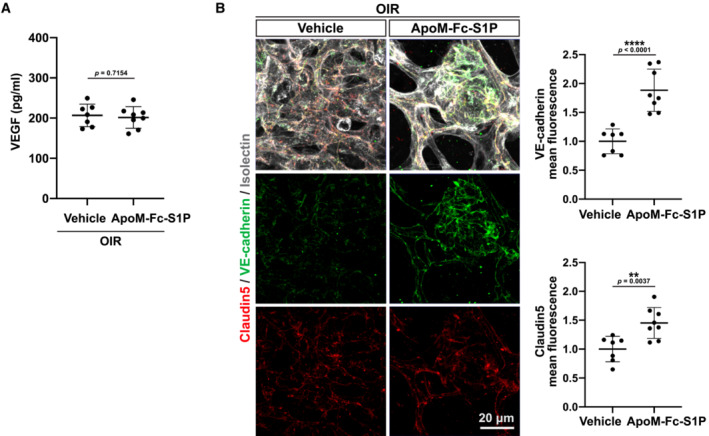
Effects of ApoM‐Fc‐S1P treatment on post‐OIR retinas VEGF expression level in P17 retinas quantified by ELISA. Retinas from post‐OIR vehicle‐ or ApoM‐Fc‐S1P‐treated (*n* = 7 and 8, respectively) WT pups at P17.Retinal flat mounts from OIR vehicle‐ or ApoM‐Fc‐S1P‐treated pups. High‐magnification view of neovascular tufts stained for VE‐cadherin (green) and Claudin‐5 (red). Junctional density was quantified as described by a minimum of seven animals per condition. Data information: Data in (A and B) were analyzed by one‐tailed Student's *t*‐test. Data are expressed as mean ± SD.

To test if chaperone‐bound S1P impacts neovascular tufts in OIR, an injectable recombinant ApoM‐Fc fusion protein (Swendeman *et al*, 2017) loaded with S1P was administered post‐oxygen exposure in OIR (at initiation of neovascularization) to S1pr1^*f/stop/f*^ and *S1pr1* ECTG mice. ApoM‐Fc‐S1P retains its binding capacity for S1P without affecting lymphocyte egress, while displaying considerable stability *in vivo* (plasma half‐life, 93.5 h) and leading to sustained endothelial signaling (Swendeman *et al*, 2017). Systemic administration of recombinant ApoM‐Fc‐S1P at P13 and P15 post‐oxygen exposure reduced neovascular tufts formation compared to vehicle‐injected littermates at P17. Enhancing both ligand and receptor (in ECTG) resulted in further reduction in neovascular tuft formation (Fig 3D). ApoM‐Fc‐S1P treatment did not reduce VEGF expression in the retina (Fig EV3A) while increasing VE‐cadherin staining at cell–cell junctions in tufts (Fig EV3B).

Our results show that systemic treatment with S1PR1 agonists or chaperone‐bound S1P protects the endothelium during OIR without suppressing normal revascularization. S1PR1 activation could therefore represent an important alternative to intravitreal VEGF‐targeted strategies which suppress both neovascularization and revascularization (Tokunaga *et al*, 2014). In this regard, the ability of S1PR1 to restore endothelial functions could be important in patients refractory to anti‐VEGF treatments. On the other hand, much remains to be understood regarding the S1P metabolism during ROP. While the role played by lipids in retinopathies, and more specifically ROP, is increasingly acknowledged (Fu *et al*, 2015; Gantner *et al*, 2019), the mechanisms explaining the systemic and local changes in S1P levels during ROP are not well defined. An alternative to S1P therapy could be to increase S1PR1 levels in retinal endothelium via gene therapy approaches (Cepko, 2012), although the effects might be less immediate than through ligand administration. Taken together, data from this study suggest that activation of the S1PR1 pathway may be an attractive candidate for treating proliferative retinopathies at large.

## Materials and Methods

### Animals

Mice were housed in a temperature‐controlled facility on a 12 h light/dark cycle, in individually ventilated cages with *ad libitum* access to sterile food and water. All mouse experiments were approved by the Institutional Animal Care and Use Committee of Boston Children's Hospital and followed the ARRIVE guidelines. Transgenic mouse models used in this study are the following: *S1pr1* ^*f/f*^ (a kind gift from Dr. Richard Proia, NIDDK, NIH), *S1pr* ^*f/stop/f*^ (Jung *et al*, 2012), *Cdh5*‐*Cre* ^*ERT2*^ (a kind gift from Dr. Ralf Adams, Max Planck Institute, Sörensen *et al*, 2009), *Apolipoprotein M* transgenic (*Apom* ^*TG*^), and *Apom* knockout (*Apom* ^−/−^) mice (Christoffersen *et al*, 2011). For tamoxifen‐inducible endothelial‐specific induction of the *S1pr1* gene in mice, *S1pr1* ^*f/stop/f*/*f/stop/f*^ mouse was crossed to *Cdh5‐Cre* ^*ERT2*^ and yielded either *S1pr1* ^*f/stop/f*/+^ (herein referred to as *S1pr1* ^*f/stop/f*^ for short) or *S1pr1* ^*f/stop/f*/+^; *Cdh5‐Cre* ^*ERT2*^ (herein referred to as *S1pr1* ECTG). For tamoxifen‐inducible endothelial‐specific deletion of *S1pr1* gene in mice, *S1pr1* ^*f/f*^ mouse was crossed to *S1pr1* ^*f/f*^; *Cdh5‐Cre* ^*ERT2*^ (herein referred to as *S1pr1* ECKO).

### Mouse experiments

Oxygen‐induced retinopathy (OIR) was induced according to the protocol described previously (Smith *et al*, 1994). Briefly, pups at P7 and their nursing dam were transferred to a chamber (A‐30274‐P, Biospherix) with an oxygen concentration maintained at 75% (ProOx Model 110, Biospherix) till P12. When the pups were returned to room oxygen levels (21%), 150 μg tamoxifen (Sigma‐Aldrich, CAT#T5648) dissolved in corn oil (Sigma‐Aldrich CAT#C8267) via intraperitoneal injection was administered two times (at P12 and P14) in order to induce or delete *S1pr1* in mice. Both males and females were used, and littermates that do not bear *Cdh5‐Cre* ^*ERT2*^ gene were used as tamoxifen‐treated controls. Mice were sacrificed and eyes were collected at the time indicated. The weights from the pups at P7 and P17 are presented in Appendix Fig S1.

To address pharmacological S1PR1 agonism upon OIR challenge, mice were given intraperitoneal injection of ApoM‐Fc‐S1P (100 μg/mouse, 4 mg/kg) at P13 and P15. SAR247799 (30 mg/kg), a S1PR1‐specific agonist, was dissolved in saline, and administered via an intraperitoneal route for 5 consecutive days (from P12 to P16). Mice were sacrificed at P17 for retinal analysis. Vehicle controls were included within the littermates in each experiment.

### Immunofluorescence staining

Whole‐mount staining of retinas was performed as previously described (Gaengel *et al*, 2012; Jung *et al*, 2012). Briefly, eyes were post‐fixed in 4% PFA in PBS at room temperature for 30 min and retinas were dissected. For cryosections, eyes were immersed in 30% sucrose in PBS overnight at 4°C, embedded in a 1:1 mixture of 30% sucrose PBS:OCT over dry ice, and then sectioned at 30 μm intervals using a cryostat (Leica Biosystems). Retinas were then permeabilized in PBLec (1% Triton X‐100 100 μM CaCl_2_, 100 μM MgCl_2_, and MnCl_2_ in PBS (pH 6.8)) at room temperature for 30 min, blocked with 1% bovine serum albumin (BSA, Sigma‐Aldrich, CAT#A6003)/PBLec at room temperature for 30 min. Primary antibodies were diluted in 1% BSA/PBLec and incubated at 4°C overnight. After thorough washing in PBLec, fluorophore‐conjugated or secondary antibodies were added to retinal tissue. Both primary and secondary antibodies used were anti‐CD31 (1:300, R&D systems, CAT#AF3628), anti‐CD45 (1:300, R&D systems, CAT#AF114‐SP), anti‐CDH5 (1:200, R&D systems, CAT#AF1002), anti‐Claudin‐5 (1:200, Invitrogen, CAT34‐1600), anti‐collagen IV (Biorad), anti‐ERG (1:300, Abcam, CAT#ab92513), anti‐fibrinogen (1:500, Accurate Chemical, CAT#YNGMFBG), anti‐GFAP(1:300, Dako, CAT#ZO334), anti‐NG2 (1:300, Millipore, CAT#MAB5320), anti‐S1PR1 (H60, sc 25489, Santa Cruze Biotechnology), Alexa Fluor 488‐conjugated anti‐ERG (1:300, Abcam, CAT#ab196374), Alexa Fluor 647‐conjugated anti‐ERG (1:300, Abcam, CAT#ab196149), Cy3‐conjugated anti‐aSMA (Sigma‐Aldrich, CAT#C6198), Alexa Fluor 488‐ (1:500, CAT#I21411), Alexa Fluor 568‐ (1:500, CAT#I21412), or Alexa Fluor 647‐ (1:500, CAT#I32450) conjugated isolectin GS‐IB4 (all from Thermo Fisher Scientific). Retinas were mounted using Fluoromount‐G slide mounting medium (Southern Biotech, CAT#0100‐01).

### Confocal imaging

Images were acquired using an LSM810 confocal microscope (Zeiss) equipped with an EC Plan‐Neofluar 10×/0.3, a Plan‐Apochromat 20×/0.8, a Plan‐Apochromat 40×/1.4 Oil DIC, or a Plan‐Apochromat 63×/1.40 Oil DIC objective. Images were taken using Zen2.1 software (Zeiss), and processed and quantified with Fiji (NIH). Figures were assembled using Adobe Photoshop and Illustrator.

For quantification of morphometric parameters in between retinas from *S1pr1* ^*f/stop/f*^ and *S1pr1* ECTG under normoxia, retinas from P9 *S1pr1* ^*f/stop/f*^ mice were used as controls.

Tufts, avascular, and total retina areas were quantified with ImageJ software (https://imagej.nih.gov/ij/download.html) (Connor *et al*, 2009). Areas of pathological neovascularization expressed as percentage of total retinal area are shown in Appendix Figs S2 and S3.

For leakage quantification, one retinal quadrant was imaged for a minimum of five animals per genotype, and the surface of fibrinogen‐positive stain outside the isolectin‐positive vascular area was quantified over the total retinal surface. For quantification of pericyte coverage of neovascular tufts, images were taken at high resolution (40×) and over 70 tufts from three different animals per genotype were analyzed. Pericyte coverage was defined as the ratio of NG2‐positive surface over the endothelial surface of neovascular tufts, delineated by isolectin‐positive staining.

For junctional density, three high‐resolution pictures (63×) of neovascular tufts were acquired per retina, and the fluorescence intensity inside 5 × 20 × 20 μm isolectin‐positive areas was quantified.

### FACS analysis

Retinas were dissected from freshly collected eyes and digested with Liberase^TM^ (Sigma‐Aldrich, CAT#5401127001, 0.26 U/ml) and deoxyribonuclease I (Sigma‐Aldrich, CAT#D4527, 10 mg/ml) in PBS (1 ml per one retina) at PBS at 37°C for 30 min. The reaction was stopped by adding SVF (final 1%) and centrifuged at 400 × *g* at 4°C for 10 min. The pellet was further washed once with FACS buffer (0.5% BSA/0.5 mM EDTA in PBS) and stained with APC‐conjugated anti‐mouse CD45 (1:300, BioLegend, CAT#103112), BV510‐conjugated anti‐mouse CD11b (1:300, BioLegend, #CAT101263), and PE‐conjugated anti‐mouse Ly6G (1:300, BD Pharmingen, #CAT5551461) antibodies for 1 h on ice. The retinal cells were washed twice and treated with DAPI to exclude dead cells. CD31^+^/CD45^−^/TER119^−^ cells were analyzed using BD FACSAria™ II (BD Biosciences).

### VEGF ELISA

Retinas were dissected from freshly collected eyes and Dounce homogenized in 200 μl PBS (supplemented with 200 mM NaCl and 1% complete protease inhibitor, Roche), then centrifuged (9,300 *g*, 20 min). Protein concentration in the supernatant was measured by BCA and 40 μg of lysate was assayed for VEGF following manufacturer's instructions (ELISA MMV00, R&D).

### Single‐cell RNAseq analysis

Data were accessed from NCBI's Gene Expression Omnibus (accession nos. GSE150703 and GSE141440). Downstream processing of the gene expression matrix was performed using the “Seurat” R package. Clustering followed by marker gene analysis enabled annotation of canonical retinal cell types. Differences in gene expression frequency and intensity are visualized using the DotPlot and FeaturePlot functions.

### Statistical analysis

Mice were analyzed regardless of their gender. OIR experiments for which the neovascular area at P17 in WT animals was under 10% of total retinal surface were excluded. Experiments were not randomized, and analysis was done in a non‐blinded fashion. Data are expressed as mean ± SD. Statistical analyses were performed unblinded using GraphPad Prism software v.8.0. In datasets containing two distinct groups, statistical comparisons were performed with the Student's *t*‐test, and *P* < 0.05 was considered statistically significant. In dataset containing three distinct groups, statistical comparisons among groups were performed using one‐way ANOVA followed by Tukey's *post‐hoc* test and *P* < 0.05 was considered statistically significant. On the figures, the error bars represent SD, and *P* < 0.05 is represented as *, *P* < 0.001 as **, *P* < 0.0001 as ***, and *P* < 0.0001 as ****. Number of animals represents biological replicates.

## Author contributions

**Colin Niaudet:** Conceptualization; formal analysis; validation; investigation; visualization; methodology; writing – original draft; writing – review and editing. **Bongnam Jung:** Validation; investigation; visualization; methodology; writing – review and editing. **Andrew Kuo:** Investigation; writing – review and editing. **Steven Swendeman:** Investigation; writing – review and editing. **Edward Bull:** Investigation; writing – review and editing. **Takahiro Seno:** Investigation; writing – review and editing. **Reed Crocker:** Software; investigation; writing – review and editing. **Zhongjie Fu:** Methodology; writing – review and editing. **Lois E H Smith:** Conceptualization; funding acquisition; writing – review and editing. **Timothy Hla:** Conceptualization; resources; supervision; funding acquisition; writing – original draft; writing – review and editing.

## Disclosure and competing interests statement

Three authors (TH, LEHS, and SS) are inventors in the patent applications for ApoM‐Fc‐S1P (WO2018052615A1).

## Supporting information



Appendix

Expanded View Figures PDF

PDF+

## Data Availability

Unprocessed microscopy images are deposited at https://doi.org/10.5281/zenodo.7651811.
