# Supplementary material for: Therapeutic activation of endothelial sphingosine‐1‐phosphate receptor 1 by chaperone‐bound S1P suppresses proliferative retinal neovascularization
Source: EMBO Mol Med. 2023 Mar 13;15(5):e16645. doi: 10.15252/emmm.202216645 (PMC10165359; doi:10.15252/emmm.202216645)
Supplement: Supplementary file 1 — Appendix [file EMMM-15-e16645-s003.pdf]

Appendix

Table of content:

Figure S1: Body weight of pups at P7 and P17
Figure S2: Total neovascular area in retina
Figure S3: Average neovascular area in retina

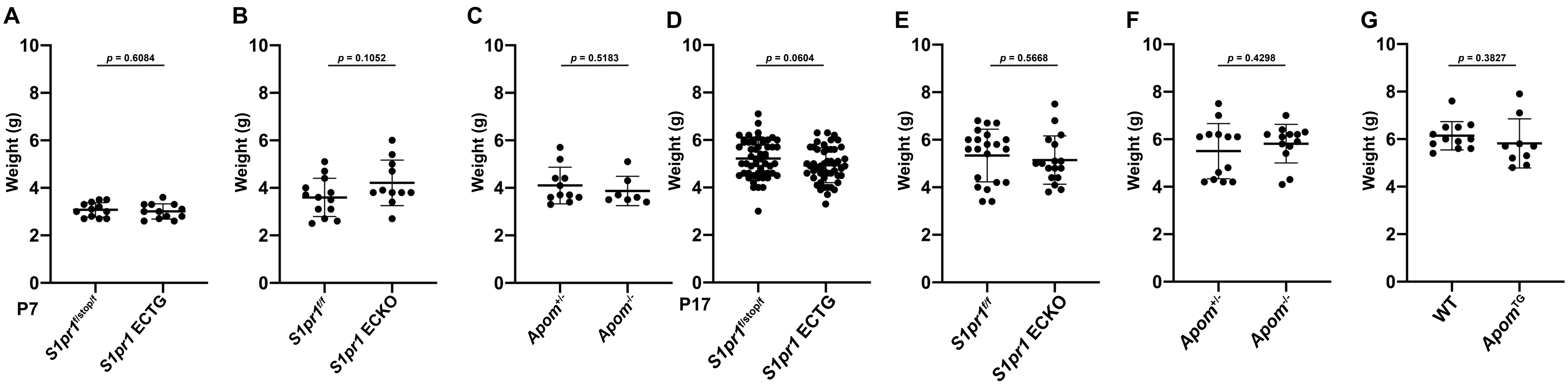

Appendix Figure S1.

(A) Body weight from *S1pr1*$^{f/stop/f}$ and *S1pr1* ECTG pups at P7
(B) Body weight from *S1pr1*$^{f/f}$ and *S1pr1* ECKO pups at P7
(C) Body weight from *Apom*$^{+/-}$ and *Apom*$^{-/-}$ pups at P7
(D) Body weight from *S1pr1*$^{f/stop/f}$ and *S1pr1* ECTG pups at P17
(E) Body weight from *S1pr1*$^{f/f}$ and *S1pr1* ECKO pups at P17
(F) Body weight from *Apom*$^{+/-}$ and *Apom*$^{-/-}$ pups at P17
(G) Body weight from WT and *Apom*^{TG} pups at P17
Data information: Data are expressed as mean ± SD. Data were analyzed by one-tailed Student's *t* test.
A minimum of 10 pups per group were analyzed.

SAR24

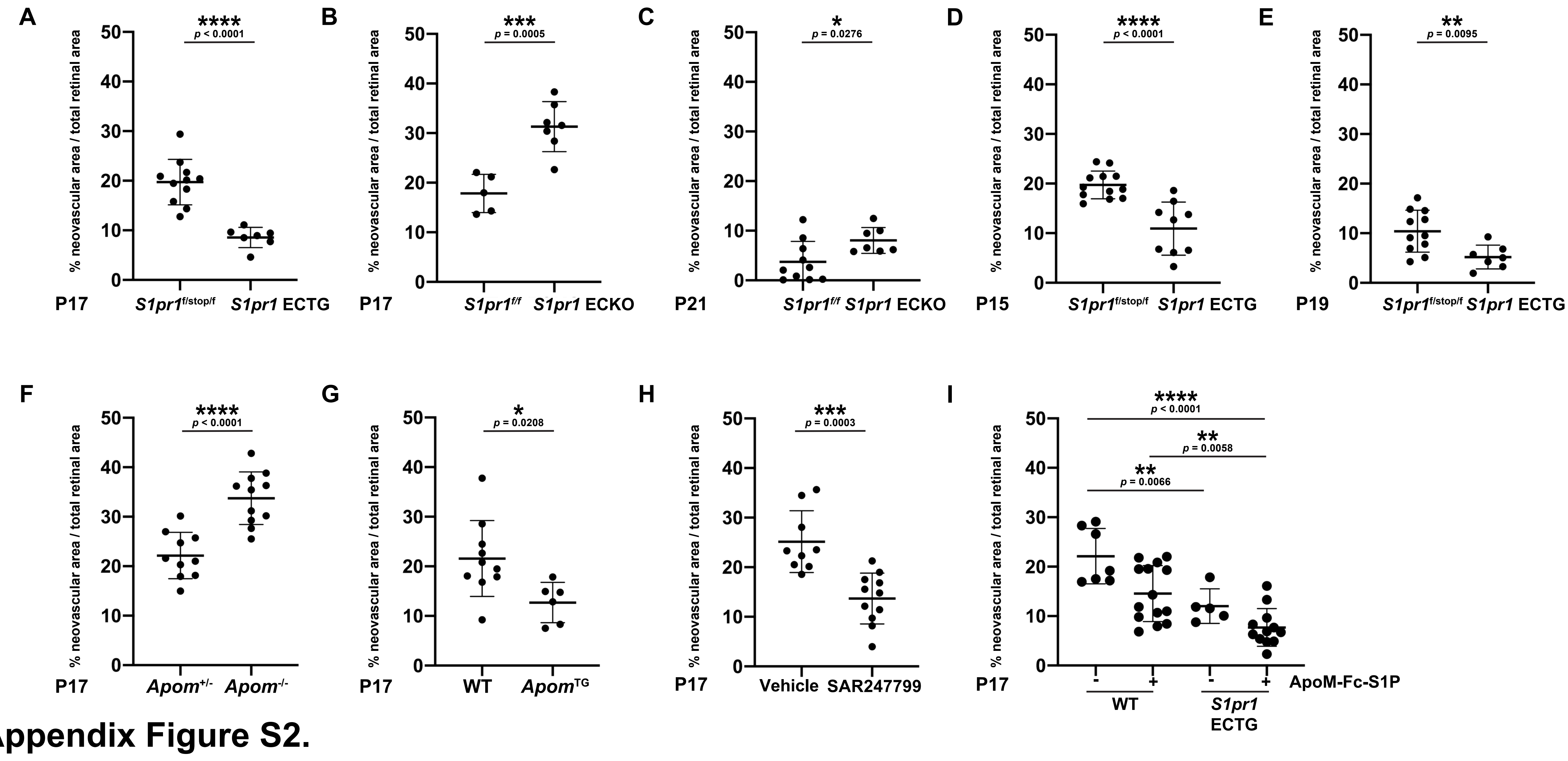

Appendix Figure S2.

(A) Quantification of total retinal neovascularization area in *S1pr1*$^{f/stop/f}$ and *S1pr1* ECTG pups at P17 after OIR
(B) Quantification of total retinal neovascularization area in *S1pr1*$^{f/f}$ and *S1pr1* ECKO pups at P17 after OIR
(C) Quantification of total retinal neovascularization area in *S1pr1*$^{f/f}$ and *S1pr1* ECKO pups at P21 after OIR
(D) Quantification of total retinal neovascularization area in *S1pr1*$^{f/stop/f}$ and *S1pr1* ECTG pups at P15 after OIR
(E) Quantification of total retinal neovascularization area in *S1pr1*$^{f/stop/f}$ and *S1pr1* ECTG pups at P19 after OIR
(F) Quantification of total retinal neovascularization area in *Apom*$^{+/-}$ and *Apom*$^{-/-}$ pups at P17 after OIR
(G) Quantification of total retinal neovascularization area in WT and *Apom*^{TG} pups at P17 after OIR
(H) Quantification of total retinal neovascularization area in SAR247799-treated, WT pups at P17 after OIR
(I) Quantification of total retinal neovascularization area in ApoM-Fc-S1P-treated, *S1pr1*$^{f/stop/f}$ and *S1pr1* ECTG pups at P17 after OIR
Data information: Data are expressed as mean ± SD. Data in A to H were analyzed by one-tailed Student's *t* test, and in I by ANOVA.
A minimum of 10 pups per group were analyzed.

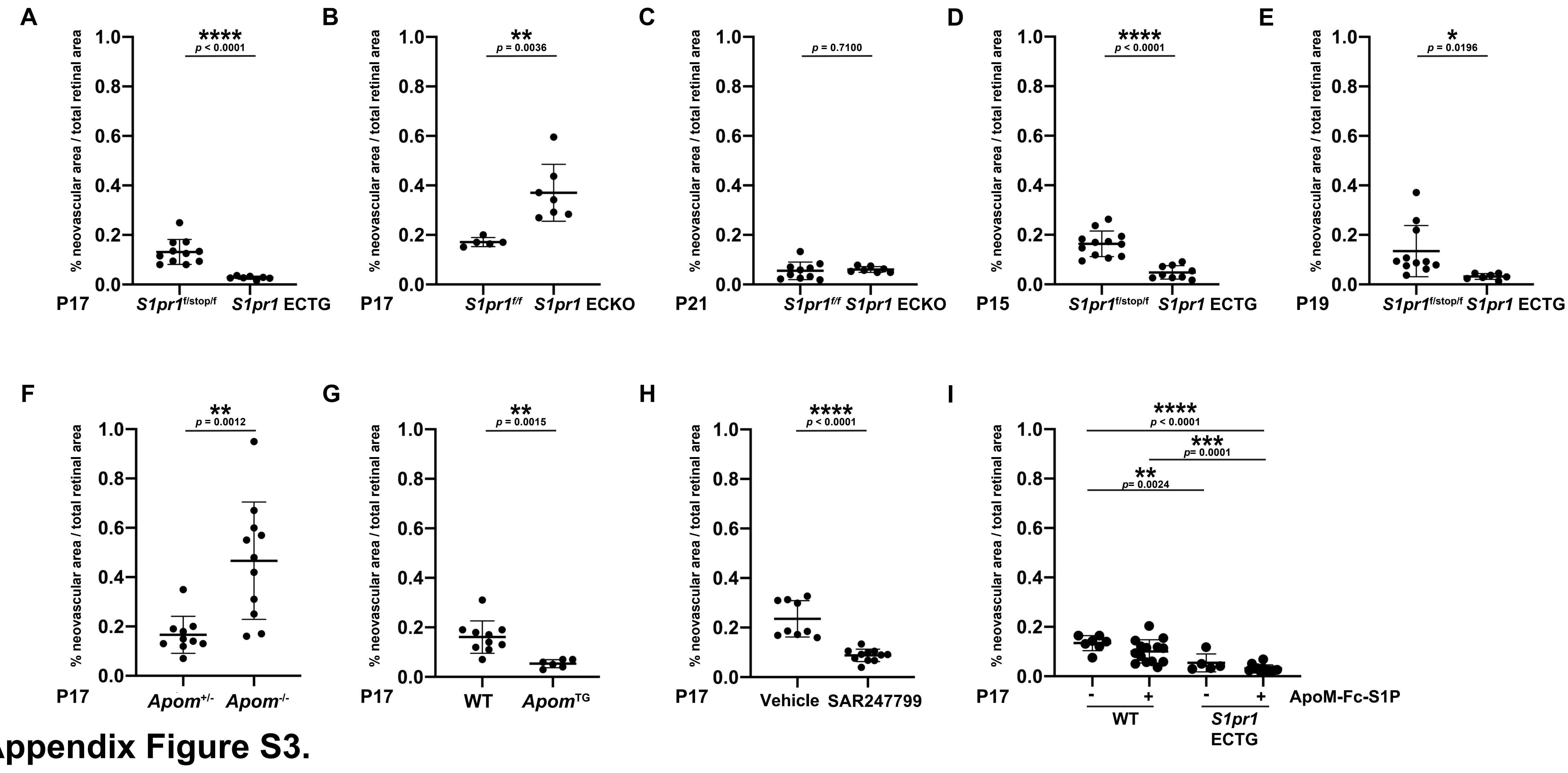

Appendix Figure S3.

(A) Quantification of average retinal neovascularization area in *S1pr1*$^{f/stop/f}$ and *S1pr1* ECTG pups at P17 after OIR
(B) Quantification of average retinal neovascularization area in *S1pr1*$^{f/f}$ and *S1pr1* ECKO pups at P17 after OIR
(C) Quantification of average retinal neovascularization area in *S1pr1*$^{f/f}$ and *S1pr1* ECKO pups at P21 after OIR
(D) Quantification of average retinal neovascularization area in *S1pr1*$^{f/stop/f}$ and *S1pr1* ECTG pups at P15 after OIR
(E) Quantification of average retinal neovascularization area in *S1pr1*$^{f/stop/f}$ and *S1pr1* ECTG pups at P19 after OIR
(F) Quantification of average retinal neovascularization area in *Apom*$^{+/-}$ and *Apom*$^{-/-}$ pups at P17 after OIR
(G) Quantification of average retinal neovascularization area in WT and *Apom*^{TG} pups at P17 after OIR
(H) Quantification of average retinal neovascularization area in SAR247799-treated, WT pups at P17 after OIR
(I) Quantification of average retinal neovascularization area in ApoM-Fc-S1P-treated, *S1pr1*$^{f/stop/f}$ and *S1pr1* ECTG pups at P17 after OIR
Data information: Data are expressed as mean ± SD. Data in A to H were analyzed by one-tailed Student's *t* test, and in I by ANOVA.
A minimum of 10 pups per group were analyzed
