# Supplementary material for: Therapeutic activation of endothelial sphingosine‐1‐phosphate receptor 1 by chaperone‐bound S1P suppresses proliferative retinal neovascularization
Source: EMBO Mol Med. 2023 Mar 13;15(5):e16645. doi: 10.15252/emmm.202216645 (PMC10165359; doi:10.15252/emmm.202216645)
Supplement: Supplementary file 2 — Expanded View Figures PDF [file EMMM-15-e16645-s002.pdf]

Expanded View Figures

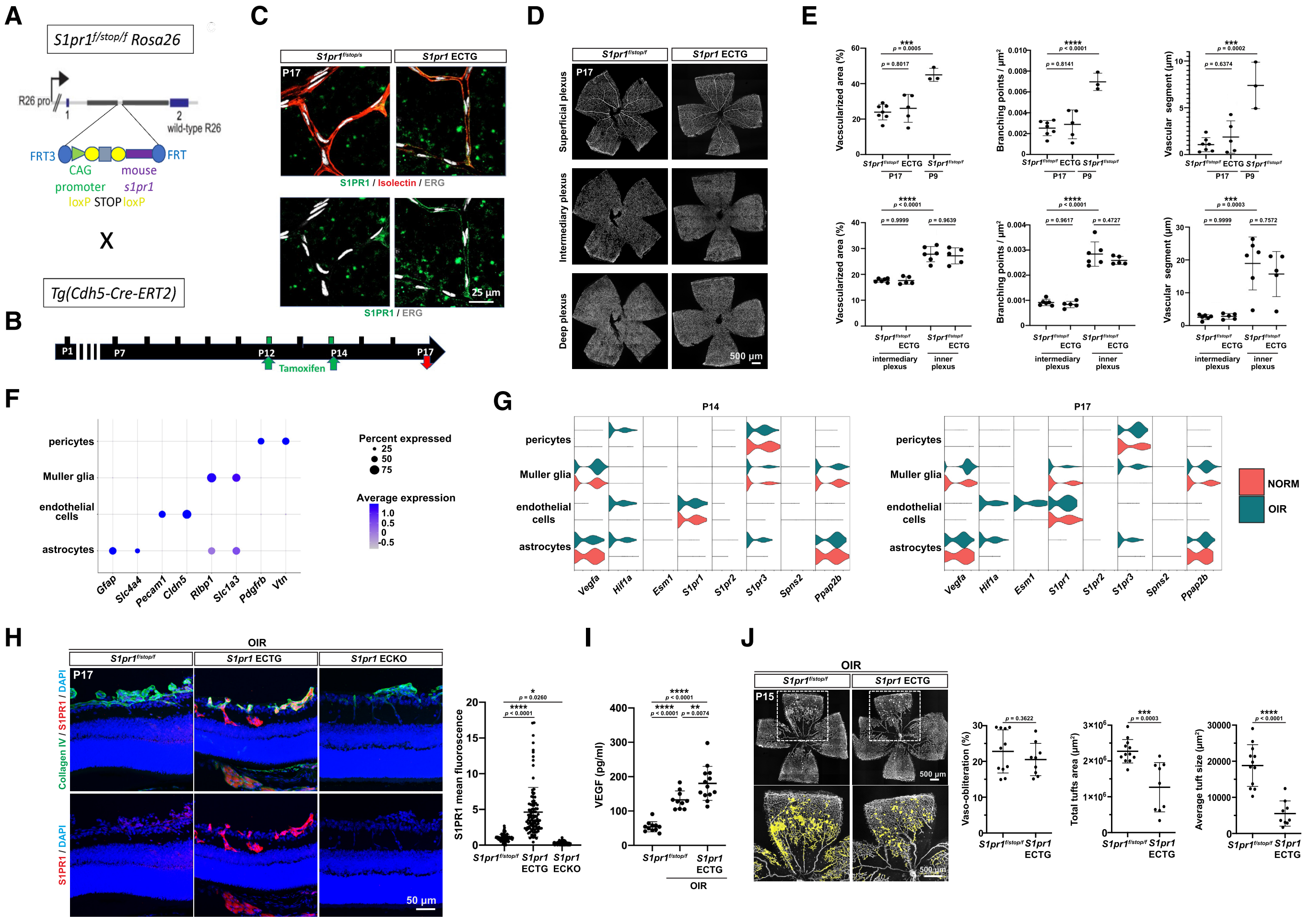

Figure EV1. S1PR1 signaling in retinal EC inhibits neovascularization in OIR.

A Schematic representation of the breeding strategy to establish *S1pr1* ECTG: females carrying the *(ROSA)26Sortm1(CAG-S1pr1)* transgene were crossed to *Cdh5-Cre*^{ERT2} Cre males.

B Strategy to induce expression of S1PR1 in post-natal endothelium. Pups were given tamoxifen at P12 and P14, and retinas were analyzed at P17.

C Flat-mounted retinas from OIR *S1pr1*$^{f/stop/f}$ and *S1pr1* ECTG pups at P17. High-magnification pictures of superficial capillaries showing S1PR1 induction in the *S1pr1* ECTG.

D Flat-mounted retinas from OIR *S1pr1*$^{f/stop/f}$ and *S1pr1* ECTG pups at P17. Retinal vasculature parameters were stained with isolectin and images of superficial (top panel), intermediary (middle panel), and deep (lower panel) plexuses are shown.

E Quantification of morphometric parameters in between retinas from *S1pr1*$^{f/stop/f}$ and *S1pr1* ECTG. Retinas from P9 S1pr1$^{f/stop/f}$ mice were used as controls.

F Dot plot representing expression level and frequency of cell types markers among non-neuronal retinal cells at P14 and P17 in OIR.

G Volcano plot showing expression level and frequency of OIR-induced genes (left) and S1P-related genes (right) among non-neuronal retinal cells at P14 (left) and P17 (right) in OIR. *S1pr4* and *S1pr5* expression was limited to a low number of endothelial cells.

H Cross sections from OIR *S1pr1*$^{f/stop/f}$, *S1pr1* ECTG, and *S1pr1* ECKO P17 pups stained for S1PR1 (red). Blood vessels are delineated by collagen IV (green) and nuclei are stained with Hoechst (blue) (left). Quantification of associated S1PR1 fluorescence inside blood vessels (right).

I VEGF level in P17 retinas quantified by ELISA. Retinas from normoxic *S1pr1*$^{f/stop/f}$ animal ($n = 10$) or OIR *S1pr1*$^{f/stop/f}$ ($n = 10$) and *S1pr1* ECTG pups ($n = 13$) at P17 were used.

J Flat-mounted retinas from OIR *S1pr1*$^{f/stop/f}$ and *S1pr1* ECTG pups at P15. Blood vessels are stained with isolectin (left). Avascular area, total neovascular tuft area, and average neovascular tuft size are quantified (right).

Data information: Data in (E, H and I) were analyzed by ANOVA test, and in (J) by one-tailed Student's *t*-test. Data are expressed as mean ± SD. A minimum of three pups per group were analyzed.

 © 2023 The Authors

Figure EV2. Neovascular tuft phenotypes in *S1pr1* ECTG at P17.

A Flat-mount view of neovascular tufts from *S1pr1*$^{f/stop/f}$, *S1pr1* ECTG, or ECKO at P17, stained for endothelial nuclei (ERG), blood vessel (isolectin), and associated endothelial nuclei quantification.

B Flat-mount view of neovascular tufts from *S1pr1*$^{f/stop/f}$ or *S1pr1* ECTG at P17, stained for immune cells (CD45), blood vessels (isolectin) (left), and FACS on retinal single-cell preparations (right). Quantification of immune cells by FACS in OIR retinas from *S1pr1*$^{f/stop/f}$ and *S1pr1* ECTG at P17. Total immune cells (CD45 positive, left panel), monocytes/macrophages (CD11b positive, middle panel), and neutrophils (CD11b and Ly6G double positive, right panel) are presented.

C Flat-mounted retinas from OIR *S1pr1*$^{f/stop/f}$ and *S1pr1* ECTG pups at P19 stained with isolectin. Avascular area and total and average neovascular tuft areas are quantified.

Data information: Data in (A) were analyzed by ANOVA, and in (B, C) by one-tailed Student's *t*-test. Data are expressed as mean ± SD. A minimum of three pups per group were analyzed.

© 2023 The Authors

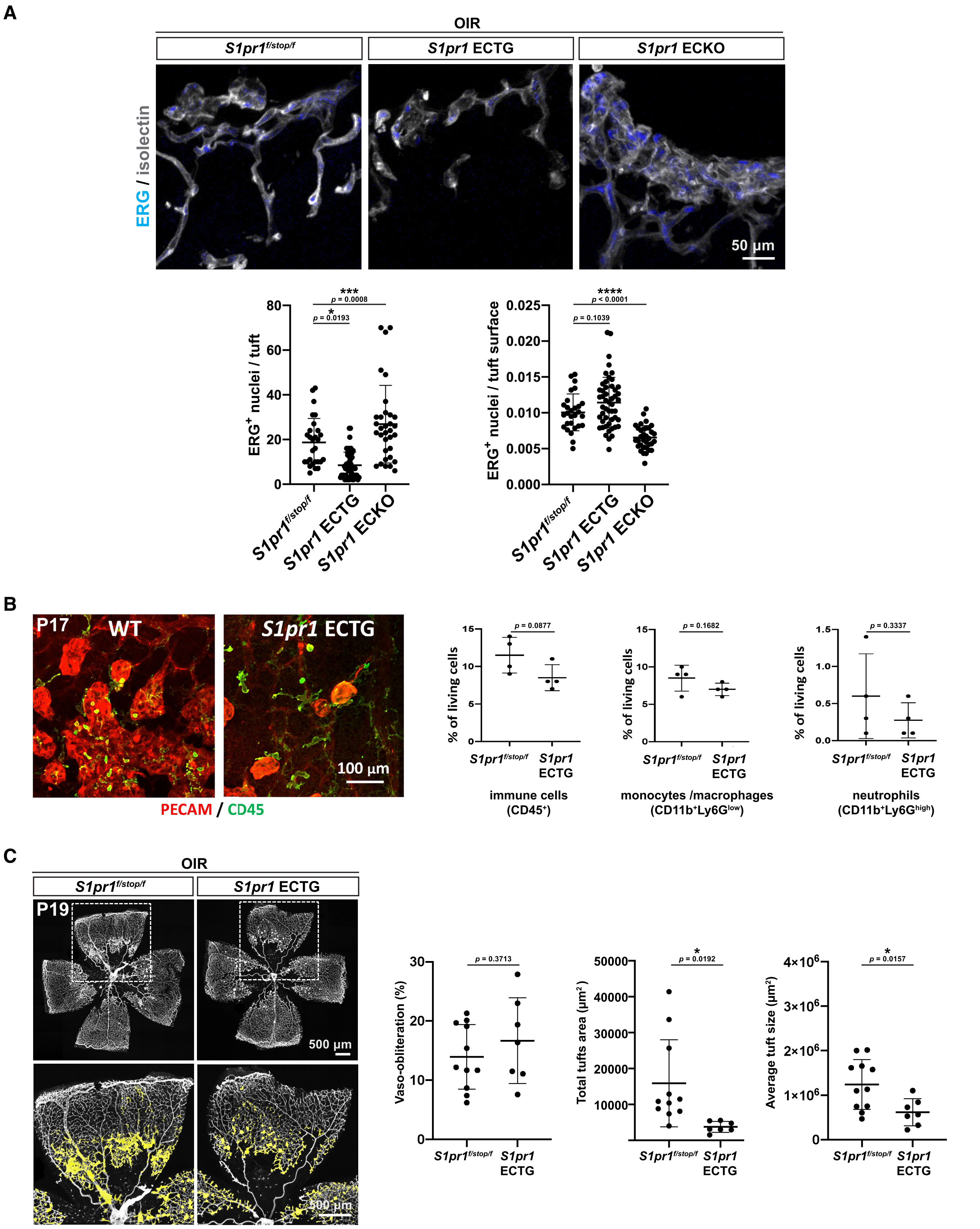

Figure EV2.

 © 2023 The Authors

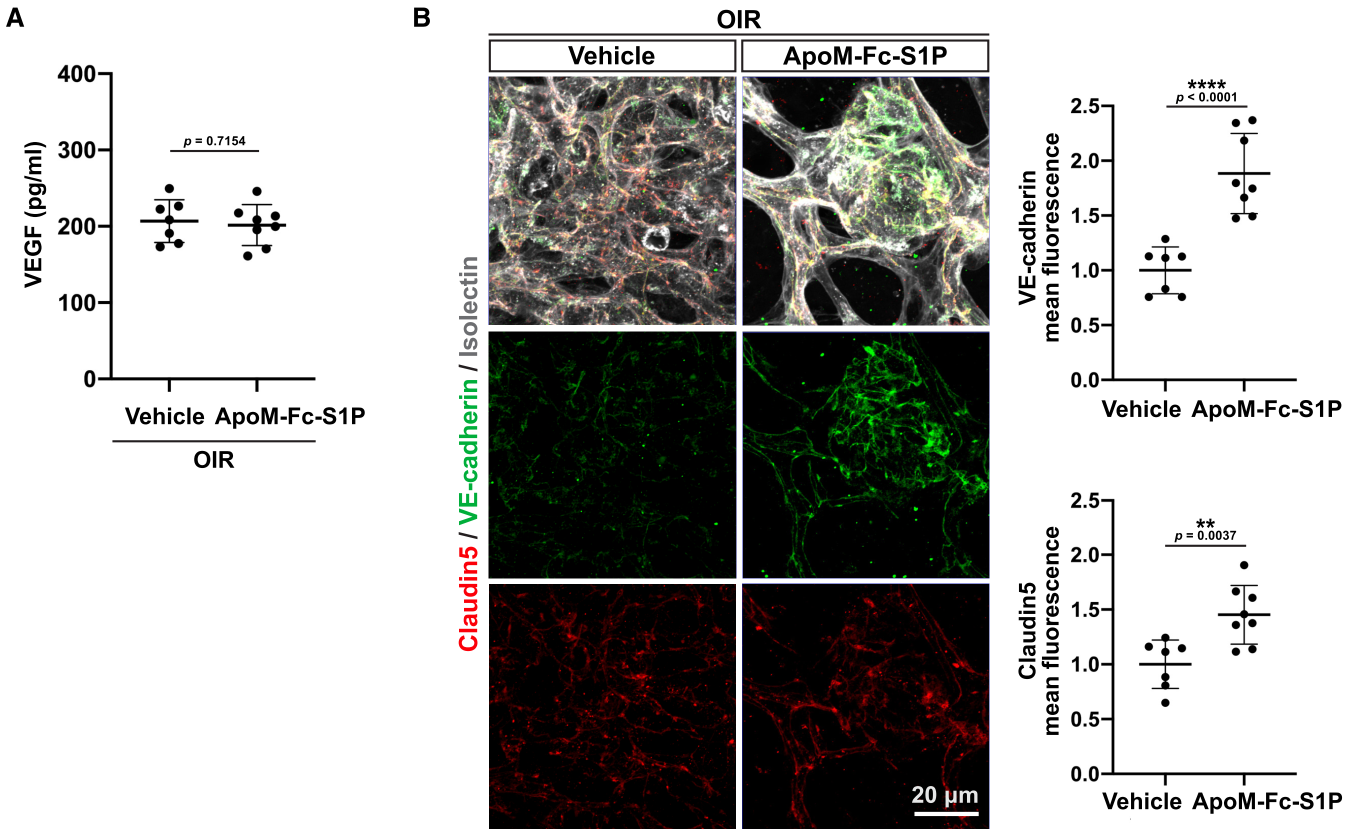

Figure EV3. Effects of ApoM-Fc-S1P treatment on post-OIR retinas.

A VEGF expression level in P17 retinas quantified by ELISA. Retinas from post-OIR vehicle- or ApoM-Fc-S1P-treated (n = 7 and 8, respectively) WT pups at P17.

B Retinal flat mounts from OIR vehicle- or ApoM-Fc-S1P-treated pups. High-magnification view of neovascular tufts stained for VE-cadherin (green) and Claudin-5 (red). Junctional density was quantified as described by a minimum of seven animals per condition.

Data information: Data in (A and B) were analyzed by one-tailed Student's t-test. Data are expressed as mean ± SD.

© 2023 The Authors
