# Supplementary material for: Therapeutic activation of endothelial sphingosine‐1‐phosphate receptor 1 by chaperone‐bound S1P suppresses proliferative retinal neovascularization
Source: EMBO Mol Med. 2023 Mar 13;15(5):e16645. doi: 10.15252/emmm.202216645 (PMC10165359; doi:10.15252/emmm.202216645)
Supplement: Supplementary file 4 [file EMMM-15-e16645-s004.pdf]

EMBO Molecular Medicine

Therapeutic activation of endothelial S1PR1 suppresses proliferative retinal neovascularization

Colin Niaudet, Bongnam Jung, Andrew Kuo, Steven Swendeman, Edward Bull, Takahiro Seno, Reed Crocker, Zhongjie Fu, Lois Smith, and Timothy Hla
DOI: 10.15252/emmm.202216645

Corresponding author: Colin Niaudet (colin.niaudet@gmail.com)

Review Timeline:

Submission Date:	25th Jul 22
Editorial Decision:	7th Sep 22
Revision Received:	4th Jan 23
Editorial Decision:	31st Jan 23
Revision Received:	15th Feb 23
Accepted:	20th Feb 23

Editor: Lise Roth

Transaction Report:

(Note: With the exception of the correction of typographical or spelling errors that could be a source of ambiguity, letters and reports are not edited. Depending on transfer agreements, referee reports obtained elsewhere may or may not be included in this compilation. Referee reports are anonymous unless the Referee chooses to sign their reports.)

7th Sep 2022

Dear Dr. Niaudet,

Thank you for the submission of your manuscript to EMBO Molecular Medicine, and please accept my apologies for the delay in getting back to you in this holiday season. We have now received feedback from the three reviewers who agreed to evaluate your manuscript. As you will see from the reports below, the referees acknowledge the interest of the study and are overall supporting publication of your work pending appropriate revisions. It will be particularly important to discuss the challenges in clinical translation based on trials conducted so far.

Addressing the reviewers' concerns in full will be necessary for further considering the manuscript in our journal, and acceptance of the manuscript will entail a second round of review. EMBO Molecular Medicine encourages a single round of revision only and therefore, acceptance or rejection of the manuscript will depend on the completeness of your responses included in the next, final version of the manuscript. For this reason, and to save you from any frustrations in the end, I would strongly advise against returning an incomplete revision.

Revised manuscripts should be submitted within three months of a request for revision; they will otherwise be treated as new submissions, except under exceptional circumstances in which a short extension is obtained from the editor.

When submitting your revised manuscript, please carefully review the instructions that follow below. We perform an initial quality control of all revised manuscripts before re-review; failure to include requested items will delay the evaluation of your revision.

We require:

1) A .docx formatted version of the manuscript text (including legends for main figures, EV figures and tables). Please make sure that the changes are highlighted to be clearly visible.

2) Individual production quality figure files as .eps, .tif, .jpg (one file per figure). For guidance, download the 'Figure Guide PDF' (https://www.embopress.org/page/journal/17574684/authorguide#figureformat).

3) A .docx formatted letter INCLUDING the reviewers' reports and your detailed point-by-point responses to their comments. As part of the EMBO Press transparent editorial process, the point-by-point response is part of the Review Process File (RPF), which will be published alongside your paper.

4) A complete author checklist, which you can download from our author guidelines (https://www.embopress.org/page/journal/17574684/authorguide#submissionofrevisions). Please insert information in the checklist that is also reflected in the manuscript. The completed author checklist will also be part of the RPF.

5) It is mandatory to include a 'Data Availability' section after the Materials and Methods. Before submitting your revision, primary datasets produced in this study need to be deposited in an appropriate public database, and the accession numbers and database listed under 'Data Availability'. Please remember to provide a reviewer password if the datasets are not yet public (see https://www.embopress.org/page/journal/17574684/authorguide#dataavailability).
In case you have no data that requires deposition in a public database, please state so in this section. Note that the Data Availability Section is restricted to new primary data that are part of this study.

6) For data quantification: please specify the name of the statistical test used to generate error bars and P values, the number (n) of independent experiments (specify technical or biological replicates) underlying each data point and the test used to calculate p-values in each figure legend. The figure legends should contain a basic description of n, P and the test applied. Graphs must include a description of the bars and the error bars (s.d., s.e.m.). Please provide exact p values.

7) We would also encourage you to include the source data for figure panels that show essential data. Numerical data should be provided as individual .xls or .csv files (including a tab describing the data). For blots or microscopy, uncropped images should be submitted (using a zip archive if multiple images need to be supplied for one panel). Additional information on source data and instruction on how to label the files are available at
.

8) Our journal encourages inclusion of *data citations in the reference list* to directly cite datasets that were re-used and obtained from public databases. Data citations in the article text are distinct from normal bibliographical citations and should directly link to the database records from which the data can be accessed. In the main text, data citations are formatted as follows: "Data ref: Smith et al, 2001" or "Data ref: NCBI Sequence Read Archive PRJNA342805, 2017". In the Reference list, data citations must be labeled with "[DATASET]". A data reference must provide the database name, accession

number/identifiers and a resolvable link to the landing page from which the data can be accessed at the end of the reference. Further instructions are available at .

9) We replaced Supplementary Information with Expanded View (EV) Figures and Tables that are collapsible/expandable online. A maximum of 5 EV Figures can be typeset. EV Figures should be cited as 'Figure EV1, Figure EV2" etc... in the text and their respective legends should be included in the main text after the legends of regular figures.
- For the figures that you do NOT wish to display as Expanded View figures, they should be bundled together with their legends in a single PDF file called *Appendix*, which should start with a short Table of Content. Appendix figures should be referred to in the main text as: "Appendix Figure S1, Appendix Figure S2" etc.
- Additional Tables/Datasets should be labeled and referred to as Table EV1, Dataset EV1, etc. Legends have to be provided in a separate tab in case of .xls files. Alternatively, the legend can be supplied as a separate text file (README) and zipped together with the Table/Dataset file.
See detailed instructions here:
.

10) The paper explained: EMBO Molecular Medicine articles are accompanied by a summary of the articles to emphasize the major findings in the paper and their medical implications for the non-specialist reader. Please provide a draft summary of your article highlighting
- the medical issue you are addressing,
- the results obtained and
- their clinical impact.
This may be edited to ensure that readers understand the significance and context of the research. Please refer to any of our published articles for an example.

11) For more information: There is space at the end of each article to list relevant web links for further consultation by our readers. Could you identify some relevant ones and provide such information as well? Some examples are patient associations, relevant databases, OMIM/proteins/genes links, author's websites, etc...

12) Author contributions: CRediT has replaced the traditional author contributions section because it offers a systematic machine readable author contributions format that allows for more effective research assessment. Please remove the Authors Contributions from the manuscript and use the free text boxes beneath each contributing author's name in our system to add specific details on the author's contribution. More information is available in our guide to authors.

13) Conflict of interest: We updated our journal's competing interests policy in January 2022 and request authors to consider both actual and perceived competing interests. Please review the policy https://www.embopress.org/competing-interests and update your competing interests if necessary.

14) Every published paper now includes a 'Synopsis' to further enhance discoverability. Synopses are displayed on the journal webpage and are freely accessible to all readers. They include a short stand first (maximum of 300 characters, including space) as well as 2-5 one-sentences bullet points that summarizes the paper. Please write the bullet points to summarize the key NEW findings. They should be designed to be complementary to the abstract - i.e. not repeat the same text. We encourage inclusion of key acronyms and quantitative information (maximum of 30 words / bullet point). Please use the passive voice. Please attach these in a separate file or send them by email, we will incorporate them accordingly.

Please also suggest a striking image or visual abstract to illustrate your article as a PNG file 550 px wide x 300-600 px high.

15) As part of the EMBO Publications transparent editorial process initiative (see our Editorial at http://embomolmed.embopress.org/content/2/9/329), EMBO Molecular Medicine will publish online a Review Process File (RPF) to accompany accepted manuscripts.
In the event of acceptance, this file will be published in conjunction with your paper and will include the anonymous referee reports, your point-by-point response and all pertinent correspondence relating to the manuscript. Let us know whether you agree with the publication of the RPF and as here, if you want to remove or not any figures from it prior to publication.
Please note that the Authors checklist will be published at the end of the RPF.

EMBO Molecular Medicine has a "scooping protection" policy, whereby similar findings that are published by others during review or revision are not a criterion for rejection. Should you decide to submit a revised version, I do ask that you get in touch after three months if you have not completed it, to update us on the status.

I look forward to receiving your revised manuscript.

Yours sincerely,

Lise Roth

Lise Roth, PhD
Senior Editor
EMBO Molecular Medicine

***** Reviewer's comments *****

Referee #1 (Comments on Novelty/Model System for Author):

This is a very strong study with both loss- and gain of function genetic models and two different strategies for systemic, translatable therapies. The presentation is overall excellent. The medical area, retinopathy, needs more therapeutic development. In conclusion, this should be highly appropriate for EMBO MM.

Referee #1 (Remarks for Author):

Niaudet et al. explore the effect of loss and gain of function of S1P signaling in the endothelium in mice challenged with oxygen-induced retinopathy. While overexpression of the S1PR1 in the endothelium in a genetic model, or systemic treatment with ApoM-Fc-bound S1P or a small molecule Gi-biased S1PR1 agonist suppressed pathological neovascularization in the OIR, loss of S1PR1 aggravated the response. A potential mechanism of action is a protective effect of S1P signling on the vascular barrier. This is an impressive study with novel findings with potential for clinical translation. A few comments are given below

1. The authors show that S1PR1 (agonists or genetic models) protects the endothelium against OIR "without suppressing normal re-vascularization". This is unclear. In the first hyperoxia phase of the challenge, VEGF expression is suppressed resulting in loss of the vasculature (vaso obliteration). In the following normoxia phase, which is experienced as relative hypoxia, VEGF expression is induced, resulting in pathological neovascularization. It would simplify the reading if the authors more clearly distinguish between vessel obliteration and pathological neovascularization. Physiological neovascularization is not tested in this assay. Clearly, S1P agonists do not protect against obliteration and therefore do not act to compensate for loss of VEGF. Instead S1P-based therapies appear to efficiently counteract the effects of excess VEGF and pathological neovascularization during the normoxia phase potentially by strengthening the vascular barrier. This scheme of events is not clearly outlined/explained in the text; please clarify.

2. Are VEGF levels affected by S1P agonists (genetic or pharmacological) during the normoxia phase?

3. Per standard in the field, please provide weights of P7 pups before oxygen-exposure to make sure that differences in weight do not explain the different responses to the OIR challenge.

4. In the figures all control litters are denoted "WT" although their genetic backgrounds differ. Please define the genotypes in the figure legend. In Methods, give information on whether the controls were treated with tamoxifen.

5. The authors summarize their findings as follows: "Taken together, these results indicate that S1PR1 signaling in the EC during OIR regulates pericyte/ EC interactions and vascular barrier function thereby impacting the extent of neovascularization. Since vascular leakage is critical for the inflammatory process, these results demonstrate that EC protective and anti-inflammatory functions of S1PR1 (Galvani et al., 2015; Christensen et al., 2016) are involved in the suppression of neovascular tuft formation in OIR." However, in the OIR-challenged mice, retinal inflammation was not affected, as shown in Fig S2A and B. Please comment/revise.

Minor
1. What is the fold-induction of S1PR expression in the ECTG mice?
2. Fig S1 panels are not ordered as described in the text, see panels F and G.
3. It would be useful for the reader if the age of the mice in the point of harvesting was given in the figures, i.e. P17, P19, P21, for the different analyses.

Referee #2 (Remarks for Author):

In the present manuscript, Niaudet and colleagues report on the role of S1P Receptor 1 in the development of retinal neovascular conditions. Using gain- and loss of-function genetic approaches the authors confirm the role of spr1 in models of oxygen-induced retinopathy (OIR), similar to earlier work in tumor angiogenesis models.

The experiments seem to have been conducted carefully and with the appropriate controls and the study has certainly some interesting aspects, for example the use of a chaperon protein to deliver S1P to the vascular endothelium. However, there are issues of novelty and translational relevance here that temper the enthusiasm for the study.

The possible role of this pathway in tumor and intraocular angiogenesis has been explored for almost two decades, using a variety of genetic and pharmacological approaches. The notion that SIPR1 activation results in suppression of angiogenesis has been extensively validated by the work of the Hla lab (for example, Cartier et al PMID: 31988136) and of other groups. The idea that activating S1PR1 with small molecules suppresses pathological angiogenesis in mouse models, including retinal angiogenesis in the OIR model, is also well established (Eresch et al, PMID: 29392309; Sorenson et al PMID: 35326420).

Earlier studies reported that a humanized anti-S1P monoclonal antibody markedly inhibited tumor (Visentin et al, PMID: 16530706) and intraocular (O'Brien et al. PMID: 19509417; Xie et al, PMID: 18781584) angiogenesis in various models. This antibody, called sonepcizumab, was then tested in patients with cancer and neovascular age-related macular degeneration but apparently there were no significant benefits.

It would be helpful if the authors discussed these clinical data and at least speculated on the reasons for such lack of success in targeting angiogenesis, which contrasts with the established use of the S1P receptor activator fingolimod in the treatment of multiple sclerosis. It would be also useful if the authors discussed the advantages of the different approaches to target this pathway, in spite of the fact that the preclinical data do not appear to be particularly discriminative.

Referee #3 (Comments on Novelty/Model System for Author):

This is an excellent manuscript that can be even better if the authors expand on the questions posed in the review. It can really be the initial path towards a new therapy for retina vasculopathies with a very strong potential for improving anti-VEGF therapies.

Referee #3 (Remarks for Author):

This is a compelling and well-design study that aims to determine the therapeutic value of targeting S1P in proliferative retinal vasculopathy. The group presents well controlled and carefully quantified experiments that supports the value of using S1P as a therapy for situations associated with vasculopathy in the retina associated with prematurity (and oxygen therapy) or diabetes. The study is scholarly presented and the data fully supports the conclusions put forward. I do not have significant concerns, only a few suggestions that would significantly enhance the value of the study as presented.

Specifically:

1- S1P has been shown to affect junctional stability, in order to seek the mechanistic underlying effect: are junctional complexes reinforced when S1P is pharmacologically provided? This is functionally addressed in Figure 2A, but not structurally or molecularly addressed. Also, How is proliferation affected in the vascular tufts?

2- Can the authors discuss the 1/2 life of ApoM-Fc-bound S1P in the circulation?

3- If treatment with ApoM-Fc-bound S1P is interrupted are the tufts susceptible to growth again? In other words, for how long is the treatment protecting the retinal vasculature to the effects of hyperoxia/hypoxia cycles? This is particularly important as the authors strike a comparison between anti-VEGF therapies and S1PR activation. Also relevant would be to demonstrate these effects in a model of diabetic retinopathy.

4- Some of the retinas look a bit "bitten up", it will be nice to show some better ones, particularly in figures 2C, 3A and 3C.

***** Reviewer's comments *****

Referee #1 (Comments on Novelty/Model System for Author):

This is a very strong study with both loss- and gain of function genetic models and two different strategies for systemic, translatable therapies. The presentation is overall excellent. The medical area, retinopathy, needs more therapeutic development. In conclusion, this should be highly appropriate for EMBO MM.

We thank the referee for this positive appreciation of our work and the constructive remarks.

Referee #1 (Remarks for Author):

Niaudet et al. explore the effect of loss and gain of function of S1P signaling in the endothelium in mice challenged with oxygen-induced retinopathy. While overexpression of the S1PR1 in the endothelium in a genetic model, or systemic treatment with ApoM-Fc-bound S1P or a small molecule Gi-biased S1PR1 agonist suppressed pathological neovascularization in the OIR, loss of S1PR1 aggravated the response. A potential mechanism of action is a protective effect of S1P signling on the vascular barrier. This is an impressive study with novel findings with potential for clinical translation. A few comments are given below

1. The authors show that S1PR1 (agonists or genetic models) protects the endothelium against OIR "without suppressing normal re-vascularization". This is unclear. In the first hyperoxia phase of the challenge, VEGF expression is suppressed resulting in loss of the vasculature (vaso obliteration). In the following normoxia phase, which is experienced as relative hypoxia, VEGF expression is induced, resulting in pathological neovascularization. It would simplify the reading if the authors more clearly distinguish between vessel obliteration and pathological neovascularization. Physiological neovascularization is not tested in this assay. Clearly, S1P agonists do not protect against obliteration and therefore do not act to compensate for loss of VEGF. Instead S1P-based therapies appear to efficiently counteract the effects of excess VEGF and pathological neovascularization during the normoxia phase potentially by strengthening the vascular barrier. This scheme of events is not clearly outlined/explained in the text; please clarify.

We thank the referee for this insightful comment. Indeed, the use of “normal re-vascularization” maybe misleading. We aimed at distinguishing the centripetal revascularization that occurs toward the optical nerve, from the neovascular tufts appearing at the surface of the vascular plexus after OIR. We have now chosen the term “re-revascularization” to avoid any confusion with physiological vascularization occurring during development and in normoxic conditions.

2. Are VEGF levels affected by S1P agonists (genetic or pharmacological) during the normoxia phase?

This is indeed a critical question to understand how S1PR1 signaling protects the retina from neovascular tufts formation. It is all the more critical that we could evidence a protective effect as early as P15. We therefore quantified VEGF in retina from *S1pr1* ECTG and control littermates at P17, now included in figure SEV2I. *S1pr1* overexpression did not result in a suppression of VEGF expression after OIR. It is correlated with an increase in VEGF production, which might be attributed to a subtle delay in revascularization of the deep plexus. We also performed a similar analysis on ApoM-Fc-S1P treated WT pups after OIR. There, the treatment did not significantly alter the amount of VEGF produced in comparison with the vehicle-treated littermates. Overall, those results suggest that the S1PR1 axis acts downstream of VEGF signaling to complete the angiogenesis process.

3. Per standard in the field, please provide weights of P7 pups before oxygen-exposure to make sure that differences in weight do not explain the different responses to the OIR challenge.
We thank the referee for this comment, and have now added an Appendix 1 with the weights of the animals at P7 and P17. The Apom^{TG} weights have been published in a recent publication which suggest no change in weight (Christoffersen et al., PMID: 29298420).

4. In the figures all control litters are denoted "WT" although their genetic backgrounds differ. Please define the genotypes in the figure legend. In Methods, give information on whether the controls were treated with tamoxifen.
We thank the referee for this comment as we use a broad range of genetic models. We have now systematically replaced the ‘WT” by the exact genotype on each figure. We also edited the Methods section to clarify that controls were tamoxifen-induced too, in the case of the inducible models.

5. The authors summarize their findings as follows: "Taken together, these results indicate that S1PR1 signaling in the EC during OIR regulates pericyte/ EC interactions and vascular barrier function thereby impacting the extent of neovascularization. Since vascular leakage is critical for the inflammatory process, these results demonstrate that EC protective and anti-inflammatory functions of S1PR1 (Galvani et al., 2015; Christensen et al., 2016) are involved in the suppression of neovascular tuft formation in OIR." However, in the OIR-challenged mice, retinal inflammation was not affected, as shown in Fig S2A and B. Please comment/revise.
We agree that there was a confusion in between vascular barrier and anti-inflammatory functions and have now rephrased the sentence, emphasizing the barrier role.

Minor
1. What is the fold-induction of S1PR expression in the ECTG mice?
We have now quantified S1PR1 expression both in *S1pr1* ECTG and ECKO. The induction was about 4 folds in the endothelium of *S1pr1* ECTG while S1PR1 was barely detectable in the S1pr1 ECKO. We have integrated these quantifications in Fig EV1H.

2. Fig S1 panels are not ordered as described in the text, see panels F and G.

We have now edited the text accordingly.

3. It would be useful for the reader if the age of the mice in the point of harvesting was given in the figures, i.e. P17, P19, P21, for the different analyses.
We agree and have now systematically edited the figures to indicate the age of the mice.

Referee #2 (Remarks for Author):

In the present manuscript, Niaudet and colleagues report on the role of S1P Receptor 1 in the development of retinal neovascular conditions. Using gain- and loss of-function genetic approaches the authors confirm the role of spr1 in models of oxygen-induced retinopathy (OIR), similar to earlier work in tumor angiogenesis models.

The experiments seem to have been conducted carefully and with the appropriate controls and the study has certainly some interesting aspects, for example the use of a chaperon protein to deliver S1P to the vascular endothelium. However, there are issues of novelty and translational relevance here that temper the enthusiasm for the study.

The possible role of this pathway in tumor and intraocular angiogenesis has been explored for almost two decades, using a variety of genetic and pharmacological approaches. The notion that SIPR1 activation results in suppression of angiogenesis has been extensively validated by the work of the Hla lab (for example, Cartier et al PMID: 31988136) and of other groups. The idea that activating S1PR1 with small molecules suppresses pathological angiogenesis in mouse models, including retinal angiogenesis in the OIR model, is also well established (Eresch et al, PMID: 29392309; Sorenson et al PMID: 35326420).

Earlier studies reported that a humanized anti-S1P monoclonal antibody markedly inhibited tumor (Visentin et al, PMID: 16530706) and intraocular (O'Brien et al. PMID: 19509417; Xie et al, PMID: 18781584) angiogenesis in various models. This antibody, called sonepcizumab, was then tested in patients with cancer and neovascular age-related macular degeneration but apparently there were no significant benefits.

It would be helpful if the authors discussed these clinical data and at least speculated on the reasons for such lack of success in targeting angiogenesis, which contrasts with the established use of the S1P receptor activator fingolimod in the treatment of multiple sclerosis. It would be also useful if the authors discussed the advantages of the different approaches to target this pathway, in spite of the fact that the preclinical data do not appear to be particularly discriminative.

We thank the referee for this perspective on S1P therapeutic applications.
Previous studies have largely focused on targeting S1P, either by modulating its synthesis (Eresch et al. PMID: 29392309) or targeting it with a monoclonal antibody (Visentin et al. PMID: 16530706, Xie et al. PMID: 18781584). In these studies, S1P was considered as a detrimental factor, despite the fact that no change in retinal S1P could be evidenced in

OIR, and that low S1P in human premature newborns is associated with severe ROP (Nilsson et al. PMID: 33862236). The fact that S1P plays a dual role, acting both on immune cells trafficking and, on the endothelium, complicates the interpretation of these publications, especially in model such as CNV which are driven by immune cells recruitment (Espinosa-Heidmann et al. PMID: 12882811, Sakurai et al. PMID: 12882810). This might explain the limited efficacy of targeting S1P, including in clinical trials. In addition, we would like to point out that the monoclonal antibody against S1P did not react with ApoM-bound S1P since it was raised against the albumin-bound form. This reagent is also not well characterized in the *in vivo* studies. Target engagement, efficacy, pharmacodynamics and pharmacokinetics were poorly characterized. Therefore, we view the S1P antibody results in publications as incomplete and refrain from drawing definitive conclusions.

To circumvent this lack of specificity, our approach focuses on specific activation of endothelial S1PR1. While we acknowledge that S1PR1 has now been solidly established as restricting angiogenesis both in some developmental and pathological contexts, the role of the S1P/S1PR1 axis in retinal vasculopathies before our study remained largely debated. We consider our study important to clarify whether or not S1PR1 can be considered as a suitable target for vascular retinopathies at large. In that perspective, the choice of ROP is not just an additional example of angiogenic response, but an adequate model to solidly establish S1PR1 as a treatment for vascular retinopathies.

Only one recent study (Sorenson et al. PMID: 35326420) also tested a specific S1PR1-agonist CYM5442, using the CNV model, yet the maximal protective effect was obtained with long-term treatment with fingolimod. Fingolimod, the earliest FDA-approved molecule developed to target S1PR, is a functional antagonist of S1PR1, with a strong effect on lymphocyte trafficking, stressing once again the difficulty to tease apart endothelial and immune action of the S1P/S1PR1 axis. Our study first establishes clearly the role of endothelial S1PR1 in vascular retinopathies by modulating its expression genetically. Second, by use of two pharmacological treatments that do not impact immune cells recruitment, our results suggest that there is an unexploited space to develop endothelial specific agonists of S1PR1 against pathological neovascularization.

Referee #3 (Comments on Novelty/Model System for Author):

This is an excellent manuscript that can be even better if the authors expand on the questions posed in the review. It can really be the initial path towards a new therapy for retina vasculopathies with a very strong potential for improving anti-VEGF therapies.

Referee #3 (Remarks for Author):

This is a compelling and well-design study that aims to determine the therapeutic value of targeting S1P in proliferative retinal vasculopathy. The group presents well controlled and carefully quantified experiments that supports the value of using S1P as a therapy for situations associated with vasculopathy in the retina associated with prematurity (and oxygen therapy) or diabetes. The study is scholarly presented and the data fully

supports the conclusions put forward. I do not have significant concerns, only a few suggestions that would significantly enhance the value of the study as presented.

We thank the referee for this positive feedback on our study and the helpful suggestions, which we addressed below.

Specifically:

1- S1P has been shown to affect junctional stability, in order to seek the mechanistic underlying effect: are junctional complexes reinforced when S1P is pharmacologically provided? This is functionally addressed in Figure 2A, but not structurally or molecularly addressed. Also, How is proliferation affected in the vascular tufts?
We thank the referee, as the status of junctional complexes is indeed critical for S1PR1 action. We have now performed dedicated analysis of adherens (Cdh5) and tight (Claudin-5) junctions in our genetic and pharmacological models upon OIR. Our new results, integrated as new Fig 2A and Fig EV3B, suggest that enhancing S1PR1 signaling reinforces the adherens junctions inside neovascular tufts, while S1pr1 ECKO mice displayed a weak level of Cdh5 staining.

As for the proliferation, which is a critical driver of the formation of neovascular tufts, we have now stained retinal cross-sections of *S1pr1* ECTG animals with the EC nuclear marker ERG. The smaller tufts found in *S1pr1* ECTG animals contained significantly less EC nuclei than in their S1pr1$^{f/stop/f}$ littermates, suggesting that S1PR1 indeed blocks the progression of the tufts, possibly at the proliferation level, as we could evidence no difference in apoptosis inside the tufts (cleaved Caspase 3 staining, not shown).

2- Can the authors discuss the 1/2 life of ApoM-Fc-bound S1P in the circulation?
This is an important aspect of our strategy. The half-life of ApoM-Fc-S1P, about 93.5 hours, was described in the princeps publication (Swendeman et al. PMID: PMID: 28811382) and we have now edited the text to indicate it.

3- If treatment with ApoM-Fc-bound S1P is interrupted are the tufts susceptible to growth again? In other words, for how long is the treatment protecting the retinal vasculature to the effects of hyperoxia/hypoxia cycles? This is particularly important as the authors strike a comparison between anti-VEGF therapies and S1PR activation. Also relevant would be to demonstrate these effects in a model of diabetic retinopathy.
We thank the referee for this insightful comment. Repeated intravitreal injections and development of resistance limit efficacy of anti-VEGF strategies and would require improvement. The OIR model is unfortunately not the best suited to tackle those critical issues, as the neovascular lesions are reversible, and disappear spontaneously after P21. Development of a more severe, irreversible model of oxygen-challenge or a different model such as diabetic retinopathy would therefore be needed, and we are currently exploring those avenues for future studies.

4- Some of the retinas look a bit "bitten up", it will be nice to show some better ones, particularly in figures 2C, 3A and 3C.

What might appear as indentations are actually the avascular areas in the retinas where the revascularization process is not completed yet.

31st Jan 2023

Dear Dr. Niaudet,

Thank you for the submission of your revised manuscript to EMBO Molecular Medicine. We have now received the enclosed reports from the three referees who re-reviewed your study. As you will see, they are now supportive of publication, and we will therefore be able to accept your manuscript once the following editorial points will be addressed:

1/ As suggested by referee #1, you may want to reformat your study as a full research article, but this remains of course your decision.

2/ Main manuscript text:
- Please address the queries from our data editors in track changes mode in the main manuscript file labelled 'Data edited MS file'. Accept the current changes and keep in track changes mode any further modification.
- Please provide up to 5 keywords.
- Materials and methods:
o Animals: please indicate the housing and husbandry conditions.
o Statistical analysis: please include a statement about randomization and exclusion criteria.
- It is mandatory to include a 'Data Availability' section after the Materials and Methods. Before submitting your revision, primary datasets produced in this study need to be deposited in an appropriate public database, and the accession numbers and database listed under 'Data Availability'. In case you have no data that requires deposition in a public database, please state so in this section ("This study includes no data deposited in external repositories.").
- Please include a "Disclosure statement and competing interests" section: We updated our journal's competing interests policy in January 2022 and request authors to consider both actual and perceived competing interests. Please review the policy https://www.embopress.org/competing-interests and update your competing interests if necessary.
- Please reformat your references to have 10 authors listed before et al.
- The figure legends should be split, with the EV figure legends displayed after the main figure legends. Please remove "not shown" from the legend from Figure EV1 (you may add a description in the methods).

3/ Figures and Appendix:
- Please provide exact p values, not a range, in the figures or in their legends, including for non-significant (NS) p values.
- Figures 2D / 3A,B,C / EV1J / EV2C: Please add highlight boxes for the zoomed image.
- Appendix 1 and 2 should be merged into one PDF and renamed Appendix Figure S1 and Appendix Figure S2. The appendix files needs a table of content and the corresponding figure legends added to the file. The figures should be referenced in the main text.

4/ At EMBO Press we encourage authors to provide source data for the main figures. More information can be found at: https://www.embopress.org/page/journal/17574684/authorguide#sourcedata

5/ Checklist:
- Newly Created Materials: please fill in the right box.
- Data Availability, primary dataset: this section refers to the Data Availability section in the manuscript text.

6/ Thank you for providing a nice synopsis picture. Please reformat it to a PNG/JPEG/TIFF file 550 px wide x 300-600 px high, and make sure the text remains legible.

7/ As part of the EMBO Publications transparent editorial process initiative (see our Editorial at http://embomolmed.embopress.org/content/2/9/329), EMBO Molecular Medicine will publish online a Review Process File (RPF) to accompany accepted manuscripts.
This file will be published in conjunction with your paper and will include the anonymous referee reports, your point-by-point response and all pertinent correspondence relating to the manuscript. Let us know whether you agree with the publication of the RPF.
Please note that the Authors checklist will be published at the end of the RPF.

I look forward to receiving your revised manuscript.

Yours sincerely,

Lise Roth

Lise Roth, PhD
Senior Editor
EMBO Molecular Medicine

***** Reviewer's comments *****

Referee #1 (Comments on Novelty/Model System for Author):

This is an impressive study which has been further improved with the revision. It exploits a number of loss- and gain-of function genetic models in the mouse, as well as therapeutic regiments. This is a high-quality study.

Referee #1 (Remarks for Author):

My personal reflection is that it is a pity that there is so much important data in the supplement. I would recommend this study for a full research article in EMBO Molecular Medicine.

Referee #2 (Remarks for Author):

The authors have largely addressed my concerns. They clarified a number of important points. I believe that the manuscript is now acceptable for publication.

Referee #3 (Comments on Novelty/Model System for Author):

The study is well presented and the genetic models appropriately selected for the questions posed.

The data indicates that modulation of S1PR1 can be a valuable path towards regulation of neovascularization.

Referee #3 (Remarks for Author):

The authors have addressed all my concerns.

The authors addressed the remaining editorial issues.

20th Feb 2023

Dear Dr. Niaudet,

Thank you for providing the revised files.
I am pleased to inform you that your manuscript is accepted for publication and is now being sent to our publisher to be included in the next available issue of EMBO Molecular Medicine!

Please read below for additional IMPORTANT information regarding your article, its publication and the production process.

Congratulations on your interesting work!

With kind regards,

Lise

Lise Roth, Ph.D
Senior Editor
EMBO Molecular Medicine

Follow us on Twitter @EmboMolMed
Sign up for eTOCs at embopress.org/alertsfeeds

*** *** *** IMPORTANT INFORMATION *** *** ***

SPEED OF PUBLICATION
The journal aims for rapid publication of papers, using using the advance online publication "Early View" to expedite the process: A properly copy-edited and formatted version will be published as "Early View" after the proofs have been corrected. Please help the Editors and publisher avoid delays by providing e-mail address(es), telephone and fax numbers at which author(s) can be contacted.

Should you be planning a Press Release on your article, please get in contact with embomolmed@wiley.com as early as possible, in order to coordinate publication and release dates.

LICENSE AND PAYMENT:

All articles published in EMBO Molecular Medicine are fully open access: immediately and freely available to read, download and share.

EMBO Molecular Medicine charges an article processing charge (APC) to cover the publication costs. You, as the corresponding author for this manuscript, should have already received a quote with the article processing fee separately. Please let us know in case this quote has not been received.

Once your article is at Wiley for editorial production you will receive an email from Wiley's Author Services system, which will ask you to log in and will present you with the publication license form for completion. Within the same system the publication fee can be paid by credit card, an invoice, pro forma invoice or purchase order can be requested.

Payment of the publication charge and the signed Open Access Agreement form must be received before the article can be published online.

PROOFS

You will receive the proofs by e-mail approximately 2 weeks after all relevant files have been sent o our Production Office. Please return them within 48 hours and if there should be any problems, please contact the production office at embopressproduction@wiley.com.

Please inform us if there is likely to be any difficulty in reaching you at the above address at that time. Failure to meet our deadlines may result in a delay of publication.

All further communications concerning your paper proofs should quote reference number EMM-2022-16645-V3 and be directed to the production office at embopressproduction@wiley.com.

EMBO Press Author Checklist

Corresponding Author Name: Hla, Niaudet	
Journal Submitted to: EMBO Mol Med	
Manuscript Number:	

USEFUL LINKS FOR COMPLETING THIS FORM
The EMBO Journal - Author Guidelines
EMBO Reports - Author Guidelines
Molecular Systems Biology - Author Guidelines
EMBO Molecular Medicine - Author Guidelines

Reporting Checklist for Life Science Articles (updated January 2022)
This checklist is adapted from Materials Design Analysis Reporting (MDAR) Checklist for Authors. MDAR establishes a minimum set of requirements in transparent reporting in the life sciences (see Statement of Task: 10.31222/osf.io/9sm4x). Please follow the journal's guidelines in preparing your manuscript.
Please note that a copy of this checklist will be published alongside your article.

Abridged guidelines for figures
1. Data
The data shown in figures should satisfy the following conditions:
- the data were obtained and processed according to the field's best practice and are presented to reflect the results of the experiments in an accurate and unbiased manner.
- ideally, figure panels should include only measurements that are directly comparable to each other and obtained with the same assay.
- plots include clearly labeled error bars for independent experiments and sample sizes. Unless justified, error bars should not be shown for technical replicates.
- if n<5, the individual data points from each experiment should be plotted. Any statistical test employed should be justified.
- Source Data should be included to report the data underlying figures according to the guidelines set out in the authorship guidelines on Data Presentation.

2. Captions
Each figure caption should contain the following information, for each panel where they are relevant:
- a specification of the experimental system investigated (eg cell line, species name).
- the assay(s) and method(s) used to carry out the reported observations and measurements.
- an explicit mention of the biological and chemical entity(ies) that are being measured.
- an explicit mention of the biological and chemical entity(ies) that are altered/varied/perturbed in a controlled manner.
- the exact sample size (n) for each experimental group/condition, given as a number, not a range;
- a description of the sample collection allowing the reader to understand whether the samples represent technical or biological replicates (including how many animals, litters, cultures, etc.).
- a statement of how many times the experiment shown was independently replicated in the laboratory.
- definitions of statistical methods and measures:
  - common tests, such as t-test (please specify whether paired vs. unpaired), simple $\chi2$ tests, Wilcoxon and Mann-Whitney tests, can be unambiguously identified by name only, but more complex techniques should be described in the methods section;
  - are tests one-sided or two-sided?
  - are there adjustments for multiple comparisons?
  - exact statistical test results, e.g., P values = x but not P values < x;
  - definition of 'center values' as median or average;
  - definition of error bars as s.d. or s.e.m.

Please complete ALL of the questions below.
Select "Not Applicable" only when the requested information is not relevant for your study.

Materials

Newly Created Materials	Information included in the manuscript?	In which section is the information available? (Reagents and Tools Table, Materials and Methods, Figures, Data Availability Section)
New materials and reagents need to be available; do any restrictions apply?	Not Applicable	

Antibodies	Information included in the manuscript?	In which section is the information available? (Reagents and Tools Table, Materials and Methods, Figures, Data Availability Section)
For **antibodies** provide the following information:<br>- Commercial antibodies: RRID (if possible) or supplier name, catalogue number and or/clone number<br>- Non-commercial: RRID or citation	Yes	Materials and Methods

DNA and RNA sequences	Information included in the manuscript?	In which section is the information available? (Reagents and Tools Table, Materials and Methods, Figures, Data Availability Section)
**Short novel DNA or RNA including primers, probes**: provide the sequences.	Not Applicable	

Cell materials	Information included in the manuscript?	In which section is the information available? (Reagents and Tools Table, Materials and Methods, Figures, Data Availability Section)
**Cell lines:** Provide species information, strain. Provide accession number in repository **OR** supplier name, catalog number, clone number, and/**OR** RRID.	Not Applicable	
**Primary cultures:** Provide species, strain, sex of origin, genetic modification status.	Not Applicable	
Report if the cell lines were recently **authenticated** (e.g., by STR profiling) and tested for mycoplasma contamination.	Not Applicable	

Experimental animals	Information included in the manuscript?	In which section is the information available? (Reagents and Tools Table, Materials and Methods, Figures, Data Availability Section)
**Laboratory animals or Model organisms:** Provide species, strain, sex, age, genetic modification status. Provide accession number in repository **OR** supplier name, catalog number, clone number, **OR** RRID.	Yes	Materials and methods
**Animal observed in or captured from the field:** Provide species, sex, and age where possible.	Not Applicable	
Please detail **housing and husbandry conditions**.	Yes	Materials and methods

Plants and microbes	Information included in the manuscript?	In which section is the information available? (Reagents and Tools Table, Materials and Methods, Figures, Data Availability Section)
**Plants:** provide species and strain, ecotype and cultivar where relevant, unique accession number if available, and source (including location for collected wild specimens).	Not Applicable	
**Microbes:** provide species and strain, unique accession number if available, and source.	Not Applicable	

Human research participants	Information included in the manuscript?	In which section is the information available? (Reagents and Tools Table, Materials and Methods, Figures, Data Availability Section)
If collected and within the bounds of privacy constraints report on age, sex and gender or ethnicity for all study participants.	Not Applicable	

Core facilities	Information included in the manuscript?	In which section is the information available? (Reagents and Tools Table, Materials and Methods, Figures, Data Availability Section)
If your work benefited from core facilities, was their service mentioned in the acknowledgments section?	Not Applicable	

Design

Study protocol	Information included in the manuscript?	In which section is the information available? (Reagents and Tools Table, Materials and Methods, Figures, Data Availability Section)
If study protocol has been **pre-registered, provide DOI in the manuscript**. For clinical trials, provide the trial registration number **OR** cite DOI.	Not Applicable	
Report the **clinical trial registration number** (at ClinicalTrials.gov or equivalent), where applicable.	Not Applicable	

Laboratory protocol	Information included in the manuscript?	In which section is the information available? (Reagents and Tools Table, Materials and Methods, Figures, Data Availability Section)
Provide DOI OR other citation details if **external detailed step-by-step protocols** are available.	Not Applicable	

Experimental study design and statistics	Information included in the manuscript?	In which section is the information available? (Reagents and Tools Table, Materials and Methods, Figures, Data Availability Section)
Include a statement about **sample size** estimate even if no statistical methods were used.	Not Applicable	
Were any steps taken to minimize the effects of subjective bias when allocating animals/samples to treatment (e.g. **randomization procedure**)? If yes, have they been described?	Yes	Materials and methods
Include a statement about **blinding** even if no blinding was done.	Yes	Materials and methods
Describe **inclusion/exclusion criteria** if samples or animals were excluded from the analysis. Were the criteria pre-established?<br><br>If sample or data points were omitted from analysis, report if this was due to attrition or intentional exclusion and provide justification.	Not Applicable	
For every figure, are **statistical tests** justified as appropriate? Do the data meet the assumptions of the tests (e.g., normal distribution)? Describe any methods used to assess it. Is there an estimate of variation within each group of data? Is the variance similar between the groups that are being statistically compared?	Yes	Materials and methods

Sample definition and in-laboratory replication	Information included in the manuscript?	In which section is the information available? (Reagents and Tools Table, Materials and Methods, Figures, Data Availability Section)
In the figure legends: state number of times the experiment was **replicated** in laboratory.	Yes	Figure legends
In the figure legends: define whether data describe **technical or biological replicates**.	Yes	Figure legends

Ethics

Ethics	Information included in the manuscript?	In which section is the information available? (Reagents and Tools Table, Materials and Methods, Figures, Data Availability Section)
Studies involving **human participants**: State details of **authority granting ethics approval** (IRB or equivalent committee(s), provide reference number for approval.	Not Applicable	
Studies involving **human participants**: Include a statement confirming that **informed consent** was obtained from all subjects and that the experiments conformed to the principles set out in the WMA Declaration of Helsinki and the Department of Health and Human Services Belmont Report.	Not Applicable	
Studies involving **human participants:** For publication of **patient photos**, include a statement confirming that consent to publish was obtained.	Not Applicable	
Studies involving experimental **animals**: State details of **authority granting ethics approval** (IRB or equivalent committee(s), provide reference number for approval. Include a statement of compliance with ethical regulations.	Yes	Material and methods
Studies involving **specimen and field samples:** State if relevant **permits** obtained, provide details of authority approving study; if none were required, explain why.	Not Applicable	

Dual Use Research of Concern (DURC)	Information included in the manuscript?	In which section is the information available? (Reagents and Tools Table, Materials and Methods, Figures, Data Availability Section)
Could your study fall under dual use research restrictions? Please check biosecurity documents and list of **select agents and toxins** (CDC): https://www.selectagents.gov/sat/list.htm	Not Applicable	
If you used a select agent, is the security level of the lab appropriate and reported in the manuscript?	Not Applicable	
If a study is subject to dual use research of concern regulations, is the name of the **authority granting approval and reference number** for the regulatory approval provided in the manuscript?	Not Applicable	

Reporting

The MDAR framework recommends adoption of discipline-specific guidelines, established and endorsed through community initiatives. Journals have their own policy about requiring specific guidelines and recommendations to complement MDAR.

Adherence to community standards	Information included in the manuscript?	In which section is the information available? (Reagents and Tools Table, Materials and Methods, Figures, Data Availability Section)
State if relevant guidelines or checklists (e.g., **ICMJE, MIBBI, ARRIVE, PRISMA**) have been followed or provided.	Yes	Materials and methods
For **tumor marker prognostic studies,** we recommend that you follow the **REMARK** reporting guidelines (see link list at top right). See author guidelines, under 'Reporting Guidelines'. Please confirm you have followed these guidelines.	Not Applicable	
For **phase II and III randomized controlled trials**, please refer to the **CONSORT** flow diagram (see link list at top right) and submit the CONSORT checklist (see link list at top right) with your submission. See author guidelines, under 'Reporting Guidelines'. Please confirm you have submitted this list.	Not Applicable	

Data Availability

Data availability	Information included in the manuscript?	In which section is the information available? (Reagents and Tools Table, Materials and Methods, Figures, Data Availability Section)
Have **primary datasets** been deposited according to the journal's guidelines (see 'Data Deposition' section) and the respective accession numbers provided in the Data Availability Section?	Not Applicable	
Were **human clinical and genomic datasets** deposited in a public access-controlled repository in accordance to ethical obligations to the patients and to the applicable consent agreement?	Not Applicable	
Are **computational models** that are central and integral to a study available without restrictions in a machine-readable form? Were the relevant accession numbers or links provided?	Not Applicable	
If publicly available data were reused, provide the respective **data citations in the reference list**.	Yes	Materials and methods
